# Adaptive group-regularized logistic elastic net regression

**DOI:** 10.1093/biostatistics/kxz062

**Published:** 2019-12-30

**Authors:** Magnus M Münch, Carel F W Peeters, Aad W Van Der Vaart, Mark A Van De Wiel

**Affiliations:** 1 Department of Epidemiology & Biostatistics, Amsterdam Public Health Research Institute, Amsterdam University Medical Centers, PO Box 7057, 1007 MB Amsterdam, The Netherlands and Mathematical Institute, Leiden University, PO Box 9512, 2300 RA Leiden, The Netherlands; 2 Department of Epidemiology & Biostatistics, Amsterdam Public Health Research Institute, Amsterdam University Medical Centers, PO Box 7057, 1007 MB Amsterdam, The Netherlands; 3 Mathematical Institute, Leiden University, PO Box 9512, 2300 RA Leiden, The Netherlands; 4 Department of Epidemiology & Biostatistics, Amsterdam Public Health Research Institute, Amsterdam University Medical Centers, PO Box 7057, 1007 MB Amsterdam, The Netherlands and MRC Biostatistics Unit, University of Cambridge, Cambridge CB2 0SR, UK

**Keywords:** Empirical Bayes, High-dimensional data, Prediction, Variational Bayes

## Abstract

In high-dimensional data settings, additional information on the features is often available. Examples of such external information in omics research are: (i) $p$-values from a previous study and (ii) omics annotation. The inclusion of this information in the analysis may enhance classification performance and feature selection but is not straightforward. We propose a group-regularized (logistic) elastic net regression method, where each penalty parameter corresponds to a group of features based on the external information. The method, termed gren, makes use of the Bayesian formulation of logistic elastic net regression to estimate both the model and penalty parameters in an approximate empirical–variational Bayes framework. Simulations and applications to three cancer genomics studies and one Alzheimer metabolomics study show that, if the partitioning of the features is informative, classification performance, and feature selection are indeed enhanced.

## 1. Introduction

In cancer genomics studies one is often faced with relatively small sample sizes as compared to the number of features. Data pooling may alleviate the curse of dimensionality, but does not apply to research settings with a unique setup, and cannot integrate other sources of information. However, external information on the features (e.g. genes) is often ubiquitously available in the public domain. We aim to use this information to improve sparse prediction. The information may come in as feature groups: e.g. the chromosome on which a gene is located (24 groups), relation to a CpG island for methylation probes (around 6 groups), or membership of a known gene signature (2 groups). Alternatively, it may be continuous, such as $p$-values from an external study. We introduce a method that allows to systematically use multiple sources of such external information to improve high-dimensional prediction.

Our methodology is motivated by four recent, small $n$ clinical omics studies, discussed in detail in Section 5. The studies concern treatment response prediction for colorectal cancer patients based on sequenced microRNAs ($n$ = 88); lymph node metastasis prediction for oral cancer, using RNAseq data ($n$=133); cervical cancer diagnostics based on microRNAs ($n$=56); and Alzheimer diagnosis based on metabolomics ($n$ = 87). Several sources of information on the features were used including external $p$-values, correlation with DNA markers, the conservation status of microRNAs, and node degree in an estimated molecular network.

As basic prediction model, we use the (logistic) elastic net regression (Zou and Hastie, 2005), which combines the desirable properties of its special cases ridge (Hoerl and Kennard, 1970) and lasso regression (Tibshirani, 1996): de-correlation and feature selection. It has been demonstrated that the prediction accuracy of penalized regression can improve by the inclusion of prior knowledge on the variables. Available methods, however, either handle one source of external information only (Lee *and others*, 2017; Tai and Pan, 2007), or do not aim for sparsity (van de Wiel *and others*, 2016).

Like others, we assume the external information to be available as feature groups; for continuous information like $p$-values, we propose a simple, data-based discretization (see Section 3). At first sight, such *a priori* grouping of the features suggests the group lasso (Meier *and others*, 2008) or one of its extensions such as the group smoothly clipped absolute deviations (grSCAD) and the group minimax concave penalty (grMCP) (Huang *and others*, 2012). These methods penalize and select features at the group level. This comes with two limitations: the group lasso (i) selects entire groups instead of single features and (ii) does not penalize adaptively: all groups are penalized equally. Extensions such as the sparse group lasso (SGL) (Simon *and others*, 2013) partly deal with (i), but do not address (ii). Our way to deal with (ii) is through differential penalization. That is, each group of features receives its own penalty parameter: the group-regularized elastic net (gren). An apparent issue with differential penalization is the estimation of the penalty parameters. Naive estimation may be done by cross-validation (CV). However, CV requires re-estimation of the model over a grid, which grows exponentially with the number of penalty parameters. Consequently, it quickly becomes computationally infeasible. We therefore propose an efficient alternative: empirical–variational Bayes (VB) estimation of the penalty parameters, which corresponds to hyperparameter estimation in the Bayesian prior framework. Because of the ubiquity of binary outcome data in clinical omics research, we focus on the logistic elastic net.

Recently, Zhang *and others* (2019) introduced a method similar to ours as it also applies VB for feature selection in logistic regression. An advantage of our method, however, is the adaptive inclusion of external information on the features to aid in prediction and feature selection, as Zhang *and others* (2019) do not estimate feature- or group-specific penalty weights. Bayesian versions of support vector machines have been used in classification problems as well (Chakraborty and Guo, 2011), but these methods also lack adaptive inclusion of external information on the features. In line with the above, our proposed method is (i) data-driven: the hyperparameters are estimated from the data, (ii) adaptive: prior information is automatically weighted with respect to its informativeness, (iii) fast compared to full Bayesian analysis or CV, and (iv) easy to use: only the data and grouping of the features are required as input.

The article is structured as follows. We introduce the model in Section 2. In Section 3, we shortly discuss possible sources of co-data. In Section 4, we derive a VB approximation to the model introduced in Section 2 and use this novel approximation in the empirical Bayes (EB) estimation of multiple, group-specific penalty parameters. In Section 5, we compare the method in a simulation study, demonstrate the benefit of the approach for two data sets, and summarize results for two additional data sets. We conclude with a discussion of some of the benefits and drawbacks of the proposed gren.

## 2. Model

The outcome variables are assumed to be binary or sums of $m_i$ disjoint binary Bernoulli trials ($y_i = \sum_{l=1}^{m_i} k_l, k_l \in \{0,1\}$ for $i=1, \dots, n$). The binomial logistic model relates the responses to the $p$-dimensional covariate vectors $\mathbf{x}_i = \begin{bmatrix} x_{i1} & \cdots & x_{ip}\end{bmatrix}^{\text{T}}$ through $y_i \sim \mathcal{B} ( m_i, \text{expit} ( \mathbf{x}_i^{\text{T}} \boldsymbol{\beta} ) )$, where $\mathcal{B} (m,\upsilon)$ is the binomial distribution with the number of trials $m$ and probability $\upsilon$, and $\text{expit} ( \mathbf{x}_i^{\text{T}} \boldsymbol{\beta} ) = \exp(\mathbf{x}^{\text{T}}_i \boldsymbol{\beta})/[1 + \exp(\mathbf{x}^{\text{T}}_i \boldsymbol{\beta})]$. Throughout the rest of the article, we assume that the model matrix $\mathbf{X} = \begin{bmatrix} \mathbf{x}_1 & \cdots & \mathbf{x}_n\end{bmatrix}^{\text{T}}$ is standardized such that $\frac{1}{n}\sum_{i=1}^n x_{ij}=0$ and $\frac{1}{n}\sum_{i=1}^n x_{ij}^2=1$ for $j=1, \dots, p$.

Assume we have a partitioning of the features in $G$ groups, such that each feature belongs to one group. Let $\mathcal{G}(g)$ be the feature index set of group $g$ for $g=1, \dots, G$ and let $\lambda'_g \in \mathbb{R}_{>0}$ denote a group-specific penalty weight. In a generalized elastic net regression, the penalized likelihood is maximized to yield parameter estimates:

(2.1) \begin{equation*} \hat{\boldsymbol{\beta}} := \underset{\boldsymbol{\beta}}{\text{argmax} \,} \log \mathcal{L}(\mathbf{y} ; \boldsymbol{\beta}) - \frac{\lambda_1}{2} \sum_{g=1}^G \sqrt{\lambda'_g} \sum_{j \in \mathcal{G}(g)} | \beta_j| - \frac{\lambda_2}{2} \sum_{g=1}^G \lambda'_g \sum_{j \in \mathcal{G}(g)} \beta_j^2, \end{equation*}

where $\mathcal{L}(\mathbf{y} ; \boldsymbol{\beta})$ denotes the likelihood function of the observed data $\mathbf{y} = \begin{bmatrix} y_1 & \cdots & y_n \end{bmatrix}^{\text{T}}$, and $\lambda_1, \lambda_2 \in \mathbb{R}_{>0}$ are the ”global“ penalty parameters. From (2.1), we see that the $\lambda'_g$’s may be interpreted as penalty multipliers. Note that the regular elastic net is recovered by setting $\forall g: \lambda'_g=1$.

Throughout the following, we assume that the geometric mean of the multipliers, weighted by their respective group sizes, is one, such that the average shrinkage of the model parameters is determined by $\lambda_1$ and $\lambda_2$. That is, we calibrate the $\lambda'_g$ such that $\prod_{g=1}^G (\lambda'_g)^{|\mathcal{G}(g)|}=1$, with $|\mathcal{G}(g)|$ the number of features in group $g$. The multipliers appear in square root form in the $L_1$-norm term to ensure that penalization on the parameter level scales with the norm. The $L_1$-norm sets some of the estimates exactly to zero, thus automatically selecting features. The $L_2$-norm ensures collinearity is well-handled. Addition of the penalty terms also prevents quasi-complete separation in logistic regression, a common phenomenon in small $n$ studies.

The maximizer of the penalized likelihood in the elastic net, corresponds to the posterior mode of a Bayesian elastic model (Zou and Hastie, 2005). Li and Lin (2010) show that the elastic net prior (see Section 2 of the supplementary material available at *Biostatistics* online for details) may be written as a computationally more convenient scale mixture of normals, with mixing parameter $\boldsymbol{\tau} = \begin{bmatrix} \tau_1 & \cdots & \tau_p \end{bmatrix}^{\text{T}}$. Using this result, we write the generalized elastic net model in its Bayesian form:

(2.2a) \begin{align*} \mathbf{y}| \boldsymbol{\beta} & \sim \prod_{i=1}^n \mathcal{B} \left( m_i, \text{expit} ( \mathbf{x}^{\text{T}}_i \boldsymbol{\beta} ) \right)\!, \\ \end{align*}

(2.2b) \begin{align*} \boldsymbol{\beta} | \boldsymbol{\tau} & \sim \prod_{g=1}^G \prod_{j \in \mathcal{G}(g)} \mathcal{N} \left(0 ,\frac{1}{\lambda' _g \lambda_2} \frac{\tau_j - 1}{\tau_j} \right)\!, \\ \end{align*}

(2.2c) \begin{align*} \boldsymbol{\tau} & \sim \prod_{j=1}^p \mathcal{TG} \left( \frac{1}{2},\frac{8 \lambda_2 }{\lambda_1^2}, \left(1,\infty \right) \right)\!. \end{align*}

Here, $\mathcal{TG} ( k,\theta,( x_l,x_u) )$ denotes the truncated gamma distribution with shape $k$, scale $\theta$, and domain $(x_l,x_u)$. In this Bayesian formulation, the penalty parameters $\boldsymbol{\lambda} = \begin{bmatrix} \lambda_1 & \lambda_2 & \lambda'_1 & \cdots & \lambda'_G \end{bmatrix}^{\text{T}}$ play the role of the hyperparameters in a Bayesian hierarchical model.

## 3. External information sources

We describe possible sources of external information in omics studies that may provide the feature groups $\mathcal{G}(g)$. Firstly, there are biologically motivated partitioning of features that are easily retrieved from online repositories. These are already discretized and may be included in the analysis as-is. Examples are: (i) pathway memberships of genes, (ii) classes of metabolites (see Section 12 of the supplementary material available at *Biostatistics* online), and (iii) conservation status of microRNAs (see Section 13 of the supplementary material available at *Biostatistics* online). A second type of external information comes in the form of continuous data. Examples are: (iv) $p$-values or false discovery rates (FDRs) from a different, related study (see Section 5.2), and (v) quality scores of the features (see Section 12 of the supplementary material available at *Biostatistics* online), and (vii) the node degrees of a network estimated on the feature data (see Section 12 of the supplementary material available at *Biostatistics* online).

gren requires discretized external information, so in the case of continuous external data, some form of discretization is required. In some cases, discretization comes naturally. For example, with external data type (iv) one might consider the ”standard“ cutoffs 0.05, 0.01, and 0.001. If the choice of cutoffs is not straightforward, we propose a data-driven heuristic, which renders a relatively finer discretization grid for data-dense than for data-sparse domains. Then, adaptation to external information is more pronounced in high-density areas. We propose to fit a piecewise linear spline to the empirical cumulative distribution function of the external data and automatically select knot locations as cutoffs (Spiriti *and others*, 2013). They also provide a data-driven method to choose the number of knots, for a given maximum number of knots. The maximum number of knots should be chosen such that each group contains enough features for stable estimation of the penalty weights. As a rule of thumb, we advise at least 20 features per group.

In many practical settings, the external information will be incomplete. We suggest to use a separate group of features with missing external information. We prefer this solution to setting the penalty multipliers for this group to one, because the absence of external information might be informative from the perspective of prediction.

## 4. Estimation

### 4.1. Empirical Bayes

If the penalty parameters are known, estimation of the elastic net model parameters is feasible with small adjustments of the available algorithms (Friedman *and others*, 2010; Zou and Hastie, 2005). Determining these penalty parameters, however, is not straightforward.

In the frequentist elastic net without group-wise penalization, two main strategies are used: (i) estimate both $\lambda_1$ and $\lambda_2$ by CV over a two-dimensional grid of values (Waldron *and others*, 2011) or (ii) re-parametrize the problem in terms of penalty parameters $\alpha= \frac{\lambda_1}{2 \lambda_2 + \lambda_1}$ and $\lambda = 2 \lambda_2 + \lambda_1$, fix the proportion of $L_1$-norm penalty $\alpha$ and cross-validate the global penalty parameter $\lambda$ (Friedman *and others*, 2010). In the generalized elastic net setting, strategies (i) and (ii) imply $2 + G$ and $1 + G$ penalty parameters, respectively. $K$-fold CV over $D$ values then results in $K \cdot D^{2 + G}$ and $K \cdot D^{1 + G}$ models to estimate. Typically, $K$ is set to 5, 10, or to the number of samples $n$, while $D$ is in the order of $100$, so that even for small $G$, the number of models to estimate is very large.

In the Bayesian framework, estimation of penalty parameters may be avoided by the addition of a hyperprior to the model hierarchy. The hyperprior takes the uncertainty in the penalty parameters into account by integrating over them. This approach introduces two issues. Firstly, the choice of hyperprior is not straightforward. Many authors suggest a hyperprior from the gamma family of distributions (Alhamzawi and Ali, 2018; Kyung *and others*, 2010), but the precise parametrization of this gamma prior is not so obvious. Secondly, the correspondence between the Bayesian and frequentist elastic net is lost. This correspondence may be exploited through the automatic feature selection property of the frequentist elastic net. Endowing the penalty parameters with a hyperprior obstructs their point estimation and, consequently, impedes automatic feature selection. Therefore, to circumvent the problem of hyperprior choice and allow for feature selection by the frequentist elastic net, we propose to estimate the penalty parameters by EB.

The most formal form of EB is maximization of the marginal likelihood with respect to the hyperparameters. The resulting hyperparameter estimates are then plugged into the prior. The marginal likelihood is often introduced as a measure of model evidence given the observed data and is computed by integrating the product of likelihood and prior with respect to the model parameters. In the case of the elastic net introduced in (2.2) EB comes down to finding:

(4.3) \begin{equation*} \hat{\boldsymbol{\lambda}} := \underset{\boldsymbol{\lambda}}{\text{argmax} \,} p_{\boldsymbol{\lambda}}(\mathbf{y}) = \underset{\boldsymbol{\lambda}}{\text{argmax} \,} \int_{\boldsymbol{\beta}} \int_{\boldsymbol{\tau}} \mathcal{L} (\mathbf{y} ; \boldsymbol{\beta}) \pi_{\boldsymbol{\lambda}}(\boldsymbol{\beta} | \boldsymbol{\tau}) \pi_{\boldsymbol{\lambda}}(\boldsymbol{\tau}) \, d\boldsymbol{\beta} d\boldsymbol{\tau}. \end{equation*}

The integrals in (4.3) are intractable in the case of the elastic net. In the omics setting, the integrals are also high dimensional, in which case numerical and Monte Carlo approximation methods become tedious and computationally expensive. Moreover, Laplace approximation is known to suffer from low accuracy in many high-dimensional settings (Shun and McCullagh, 1995). In Casella (2001), an EM algorithm is described that estimates the hyperparameters. This EM algorithm iteratively maximizes the expected joint log likelihood, such that the sequence:

(4.4) \begin{equation*} \boldsymbol{\lambda}^{(k + 1)} = \underset{\boldsymbol{\lambda}}{\text{argmax} \,} \mathbb{E}_{\boldsymbol{\beta}, \boldsymbol{\tau} | \mathbf{y}} [ \log [ \mathcal{L} (\mathbf{y} ; \boldsymbol{\beta}) \pi_{\boldsymbol{\lambda}}(\boldsymbol{\beta} | \boldsymbol{\tau}) \pi_{\boldsymbol{\lambda}}(\boldsymbol{\tau}) ] | \boldsymbol{\lambda}^{(k)} ] \end{equation*}

converges to a local maximum of the marginal likelihood. The difficulty herein is in the calculation of the expected joint log likelihood. Casella (2001) suggests to approximate the expectation by its Monte Carlo expectation. Although elegant and simple, this method requires a converged MCMC sample from the posterior for every iteration: a computationally intensive procedure. Roy and Chakraborty (2017) introduce generalized importance sampling for the Bayesian elastic net, such that we just need a limited, pre-specified number of MCMC chains. However, this still requires several converged chains so is not feasible in many high-dimensional omics settings. We propose to tackle this problem by approximating the expectation in (4.4) using VB.

### 4.2. Variational Bayes

VB is a widely used method to approximate Bayesian posteriors. It has successfully been applied in a wide range of applications, including genetic association studies (Carbonetto and Stephens, 2012) and gene network reconstruction (Leday *and others*, 2017). In VB, the posterior is approximated by a tractable form and estimated by optimizing a lower bound on the marginal likelihood of this model (see Section 3 of the supplementary material available at *Biostatistics* online for the lower bound of the proposed model). For an extensive introduction and concise review, see Beal (2003) and Blei *and others* (2017).

To simplify the computations of our VB approximation, we follow Polson *and others* (2013) and introduce latent variables $\omega_i$, for $i=1, \dots, n$. Conditional on $\boldsymbol{\beta}$, the $\omega_i$ are independent of $y_i$ and Pólya-Gamma distributed (see Section 4 of the supplementary material available at *Biostatistics* online for more details). We augment Model (2.2) with:

(4.5) \begin{equation*} \boldsymbol{\omega} | \boldsymbol{\beta} \sim \prod_{i=1}^n \mathcal{PG}(m_i, |\mathbf{x}_i^{\text{T}} \boldsymbol{\beta}| )\!. \end{equation*}

Our VB approximation to the posterior distribution of (2.2) and (4.5) factorizes over blocks of parameters. We choose the blocks such that:

(4.6) \begin{equation*} p (\boldsymbol{\omega}, \boldsymbol{\beta}, \boldsymbol{\tau} | \mathbf{y}) \approx Q(\boldsymbol{\omega}, \boldsymbol{\beta}, \boldsymbol{\tau}) = q_{\boldsymbol{\omega}} (\boldsymbol{\omega}) q_{\boldsymbol{\beta}} (\boldsymbol{\beta}) q_{\boldsymbol{\tau}} (\boldsymbol{\tau}). \end{equation*}

Writing $\boldsymbol{\theta} = \begin{bmatrix} \boldsymbol{\theta}_1 & \boldsymbol{\theta}_2 & \boldsymbol{\theta}_3 \end{bmatrix} = \begin{bmatrix} \boldsymbol{\omega} & \boldsymbol{\beta} & \boldsymbol{\tau} \end{bmatrix}$, calculus of variations renders the optimal distributions $q^*_{\boldsymbol{\theta}_j} (\boldsymbol{\theta}_j) \propto \exp \{\mathbb{E}_{\boldsymbol{\theta} \backslash \boldsymbol{\theta}_j} [\log p (\boldsymbol{\theta} | \mathbf{y})]\}$, where optimality is achieved in terms of the Kullback–Leibler divergence of the posterior to the approximate distribution (Beal, 2003). The approximation in (4.6) renders both the posterior parameter calculations and the expected joint log likelihood in (4.4) tractable.

After a change of variables $\psi_j = \tau_j - 1$, we find the optimal distributions as:

(4.7) \begin{equation*} q^*_{\boldsymbol{\beta}} (\boldsymbol{\beta}) \sim \mathcal{N} (\boldsymbol{\mu}, \boldsymbol{\Sigma}) \text{, } q^*_{\boldsymbol{\omega}} (\boldsymbol{\omega}) \sim \prod_{i=1}^n \mathcal{PG} (m_i, c_i) \text{, and } q^*_{\boldsymbol{\psi}}(\boldsymbol{\psi}) \sim \prod_{j=1}^p \mathcal{GIG} (\frac{1}{2}, \frac{\lambda_1^2}{4 \lambda_2}, \chi_j)\!, \end{equation*}

where $\mathcal{GIG} (\cdot)$ denotes the generalized inverse Gaussian distribution (See Section 5 of the supplementary material available at *Biostatistics* online for the derivations). The so-called variational parameters in (4.7) contain cyclic dependencies, so we update them by (13) in Section 6 of the supplementary material available at *Biostatistics* online until convergence. Naive calculation of the variational parameters is computationally expensive. In Section 7 of the supplementary material available at *Biostatistics* online, we show that informed calculation results in a significant reduction of computational complexity.

### 4.3. Empirical-variational Bayes

VB was shown to underestimate the posterior variance of the parameters, both numerically and theoretically, in several settings (Rue *and others*, 2009; Wang and Titterington, 2005). This coincides with our experience that the global penalty parameters $\lambda_1$ and $\lambda_2$ tend to be overestimated, because they are inversely related to the posterior variances of the $\beta_j$. To prevent overestimation we use the parametrization of Friedman *and others* (2010) as discussed in Section 4.1: we fix $\alpha$ and estimate $\lambda$ by CV of the regular elastic net model, such that the overall penalization is determined by CV of only $\lambda$. By combining CV of the global penalty parameter $\lambda$ with EB estimation of the penalty multipliers $\boldsymbol{\lambda}' = \begin{bmatrix} \lambda'_1 & \cdots & \lambda'_G \end{bmatrix}^{\text{T}}$, the estimation is robust to underestimation of the VB posterior variances. For $\alpha$, Hastie and Qian (2016) recommend to either fix it *a priori*, or compare results for several choices of $\alpha$. We use the latter.

To estimate the penalty multipliers, the intractable posterior expectation in (4.4) is approximated using the VB posterior:

(4.8) \begin{equation*} \mathbb{E}_{Q} [ \log \mathcal{L}_{\boldsymbol{\lambda}'}(\mathbf{y}, \boldsymbol{\omega}, \boldsymbol{\beta}, \boldsymbol{\tau}) | \boldsymbol{\lambda}'^{(k)} ] = \frac{1}{2} \sum_{g=1}^G |\mathcal{G}(g)| \log (\lambda'_g) - \frac{(1 - \alpha) \lambda}{4} \sum_{g=1}^G \lambda'_g d^{(k)}_g + C, \end{equation*}

where $C$ is constant in $\boldsymbol{\lambda}'$ (see Section 6 of the supplementary material available at *Biostatistics* online for the full derivation and the $d^{(k)}_g$ terms). An estimate of the new penalty multipliers is now given by (14) in Section 6 of the supplementary material available at *Biostatistics* online. Although the solution to (14) in the Supplementary material available at *Biostatistics* online is not available in closed form, this convex problem is easily solved by a numerical optimization routine. The full estimation procedure is summarized in Algorithm 1.

Algorithm 1 **Group-regularized empirical Bayes elastic net**

**Require:** $\mathbf{X}, \mathbf{y}, \mathcal{G}, \alpha, \epsilon_1, \epsilon_2$

**Ensure:** $\lambda, \boldsymbol{\lambda}', \boldsymbol{\Sigma}, \boldsymbol{\mu}$

$\quad\textrm{Estimate}$ $\lambda$ by CV of the regular elastic net model

**while** $|\frac{\boldsymbol{\lambda}'^{(k + 1)} - \boldsymbol{\lambda}'^{(k)}}{\boldsymbol{\lambda}'^{(k)}}| > \epsilon_1$ **do**

**while** $\underset{i}{\max} \, |\frac{\boldsymbol{\Sigma}_{ii}^{(k+1)} - \boldsymbol{\Sigma}_{ii}^{(k)}}{\boldsymbol{\Sigma}_{ii}^{(k)}}| > \epsilon_2$ or $\underset{i}{\max} \, |\frac{\boldsymbol{\mu}_{i}^{(k+1)} - \boldsymbol{\mu}_{i}^{(k)}}{\boldsymbol{\mu}_{i}^{(k)}}| > \epsilon_2$**do**

$\quad\quad\quad\textrm{Update}$ $\boldsymbol{\Sigma}$, $\boldsymbol{\mu}$, $c_i$ for $i=1, \dots, n$ and $\chi_j$ for $j=1, \dots, p$ using (13) in the Supplementary material available at *Biostatistics* online

**end while**

$\quad\quad\textrm{Update}$ $\boldsymbol{\lambda}'$ by (14) in the Supplementary material available at *Biostatistics* online

**end while**

### 4.4. Feature selection

Feature selection is often desirable in high-dimensional prediction problems. For example, biomarker selection may lead to a large reduction in costs by supporting targeted assays. Bayesian feature selection is often done by inspection of posterior credible intervals. However, the Bayesian lasso’s (a special case of the elastic net) credible intervals are known to suffer from low frequentist coverage (Castillo *and others*, 2015). We therefore propose to select features in the frequentist paradigm.

Frequentist feature selection is trivial after estimation of the penalty multipliers. We therefore simply plug the estimated penalty parameters into some frequentist elastic net algorithm that allows for differential penalization. In our package gren, we involve the R-package glmnet (Friedman *and others*, 2010), which automatically selects features. Furthermore, to select a specific number of features, we simply adjust the global $\lambda$ to render the desired number.

## 5. Simulations and applications

### 5.1. Simulations

We conducted a simulation study in which we compared gren to the regular elastic net and ridge models, GRridge (van de Wiel *and others*, 2016), composite mimimax concave penalty (cMCP) (Breheny and Huang, 2009), and the group exponential lasso (gel) (Breheny, 2015). GRridge is similar to gren in the sense that it estimates group-specific penalty multipliers. The two main differences with gren are (i) the absence of an $L_1$-norm penalty and (ii) the estimation procedure. The other methods are extensions of the group lasso and not adaptive on the group level. However, in contrast to the original group lasso, they select single features, instead of complete groups.

We simulated data according to five different scenarios: (i) differential signal between the groups and uniformly distributed model parameters; (ii) a large number of small groups of features; (iii) no differential signal between the groups, but strong correlations within groups of features; (iv) differential signal between the groups and heavy-tailed distributed model parameters; and (v) a very sparse setting with no signal in some of the groups.

In all scenarios, the $y_i$ are sampled from the logistic model introduced in Section 2, where the $\mathbf{x}_i^{\text{T}}$ are multivariate Gaussian and $\boldsymbol{\beta}$ is scenario dependent. Area under the receiver operator curve (AUC) and Brier skill score, averaged over 100 repeats, were used to evaluate performance for models trained on $n=100$ samples and $p \approx 1000$ features. Full descriptions of the scenarios and corresponding results are given in Section 15 of the supplementary material available at *Biostatistics* online. Here, we summarize the results.

In terms of Brier skill score, the ridge methods generally outperform the elastic net methods, which in turn outperform the group lasso methods. In Scenario (i), gren and GRridge outperform the other methods in terms of AUC. In scenario (ii), the AUC follows our expectation: gren, and to a lesser extent GRridge underperform due to overfitting. The regular elastic net outperforms the group lasso methods. In Scenario (iii), gren and to a lesser extent the regular elastic net suffer from the high correlations. The regular elastic net outperforms gren, which is an indication that high correlations impair penalty parameter estimation. Scenario (iv) follows the expected pattern, with GRridge and gren outperforming their respective non-group-regularized counterparts, as well as the SGL extensions. In Scenario (v), gren outperforms all other methods, which is an indication that gren is able to pick up the sparse differential signal.

In addition to simulations from the correct model, i.e., model (2.2), we simulated from an incorrect model to investigate the performance of gren under model misspecification. We investigated both link misspecification and non-linear feature effects misspecification. The details of the simulations and detailed results are given in Section 15.7 of the supplementary material available at *Biostatistics* online. In all investigated Scenarios gren outperformed the regular elastic net in terms of predictive performance; an indication that gren is relatively robust to model misspecification.

### 5.2. Application to microRNAs in colorectal cancer

We investigated the performance of gren on data from a microRNA sequencing study (Neerincx *and others*, 2018). The aim of the study was to predict treatment response in 88 colorectal cancer patients, coded as either non-progressive/remission (70 patients) or progressive (18 patients). After pre-processing and normalization, 2114 microRNAs remained. Four unpenalized clinical covariates were included in the analysis: prior use of adjuvant therapy (binary), type of systemic treatment regimen (ternary), age, and primary tumor differentiation (binary).

In a preliminary experiment on different subjects, the microRNA expression levels of primary and metastatic colorectal tumor tissues were compared to their normal tissue counterparts (Neerincx *and others*, 2015). The two resulting FDRs were combined through the harmonic mean (Wilson, 2019) and discretized using the method described in Section 3. This yielded four groups of features: (i) $\text{FDR} \leq 0.0001$, (ii) $0.0001 < \text{FDR} \leq 0.0186$, (iii) $0.0186 < \text{FDR}$, and (iv) missing FDR. We expect that incorporation of this partitioning enhances therapy response classification, because tumor-specific microRNAs are likely to be more relevant than non-specific ones.

We compared the performance of gren to ridge, GRridge, random forest (Breiman, 2001), elastic net, SGL by Simon *and others* (2013), cMCP, and gel. Of the latter three methods, we only present the best performing one, cMCP, here. The results for SGL and gel are presented in Section 11 of the supplementary material available at *Biostatistics* online. For the methods with a tuning parameter $\alpha$, we show the best performing $\alpha$ here and refer the reader to Section 11 of the supplementary material available at *Biostatistics* online for the remaining $\alpha$’s.

**Fig. 1.**
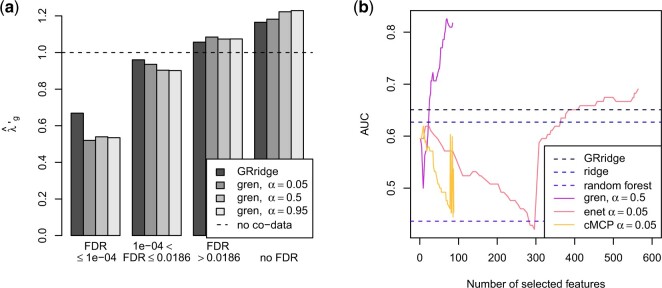
Estimated (a) penalty multipliers and (b) AUC in the colorectal cancer example.

To estimate performance, we split the data into 61 training and 27 test samples, stratified by treatment response. We estimated the models on the training data and calculated AUC on the test data. We present AUC for a range of model sizes, together with the estimated penalty multipliers for gren and GRridge in Figure 1. Brier skill scores are presented in Section 11 of the supplementary material available at *Biostatistics* online. In addition, we investigated the sensitivity of the multiplier estimation in Section 11 of the supplementary material available at *Biostatistics* online.

The estimated penalty multipliers are according to expectation: the small FDR group receives the lowest penalty, followed by the medium and large FDR groups. The missing FDR group receives the strongest penalty, thereby confirming that absence of information is informative itself. We observe that gren outperforms the other methods for smaller models. Selection of larger models is impaired by the large $\alpha$, a property inherent to $L_1$-norm penalization. With a smaller $\alpha$ larger models are possible (see Section 11 of the supplementary material available at *Biostatistics* online). The performance of cMCP is somewhat unstable. This unstable estimation is an issue in all investigated group lasso extensions. Overall, the inclusion of the extra information on the features benefits predictive performance: both GRridge and gren outperform their respective non-group-regularized versions, albeit only slightly for GRridge. The random forest performs worse here.

### 5.3. Application to RNAseq in oral cancer

The aim of this second study is to predict lymph node metastasis in oral cancer patients using sequenced RNAs from TCGA (The Cancer Genome Atlas Network, 2015). The features are 3096 transformed and normalized TCGA RNASeqv2 gene expression values for 133 HPV-negative oral tumors. Of the corresponding patients, 76 suffered from lymph node metastasis, while 57 did not. For a thorough introduction of these data, see te Beest *and others* (2017).

We considered two sources of external feature information: (i) the cis-correlations between the RNASeqv2 data and TCGA DNA copy numbers on the same patients, quantified by Kendall’s $\tau$ and binned into five groups using the rule from Section 3. In addition, we used (ii) $p$-values from an independent microarray data set described in Mes *and others* (2017), again binned into five groups. We expect features with a large positive Kendall’s $\tau$ to be more important in metastasis prediction (Masayesva *and others*, 2004). Likewise, we expect features with low external $p$-values to be more important.

**Fig. 2.**
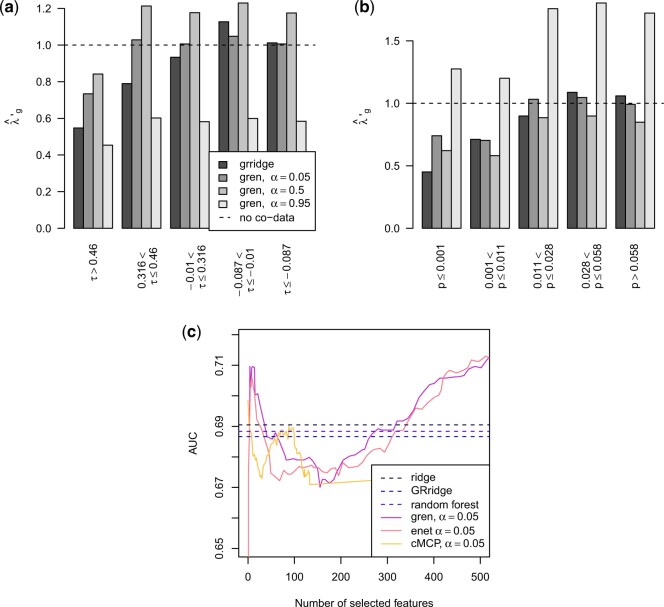
Estimated (a) penalty multipliers for the cis-correlations and (b) external $p$-values, and (c) estimated AUC in the oral cancer example.

We compared gren to the same methods as in Section 5.2. However, since the feature information consists of two partitions with overlapping groups, we used extensions of cMCP and gel that allow for overlapping groups (Zeng and Breheny, 2016). In SGL, we cross-tabulated the two partitions to create one grouping of the features. We estimated AUC on another independent validation set of 97 patients (Mes *and others*, 2017), containing microarray features, normalized to account for a scale difference with the RNAseq data. We present estimated AUC on this validation set for GRridge, ridge, random forest, gren, elastic net, and the best performing group lasso extension, cMCP, together with the estimated penalty multipliers in Figure 2. For the methods with an $\alpha$ parameter, we pick the best performing one. Results for SGL, gel, other values of $\alpha$, and the Brier skill scores are presented in Section 12 of the supplementary material available at *Biostatistics* online.

The estimated penalty multipliers follow the expected pattern: larger cis-correlations receive smaller penalties and smaller $p$-values are penalized less. The AUC of gren is slightly better than the other methods for a range of model sizes. In this example, ridge, GRridge, and random forest perform almost identical, while gren outperforms the regular elastic net.

### 5.4. Additional applications

Two additional applications are presented in Sections 13 and 14 of the supplementary material available at *Biostatistics* online. The first one is concerned with diagnosis of Alzheimer’s from 230 metabolites’ expression levels in 174 subjects. We included several sources of extra information on the metabolites. In this example, gren performs worse than the group lasso methods for larger models. This is due to the smaller number of features to learn the penalty parameters from. Additionally, many metabolites are strongly negatively correlated, which further impairs penalty parameter estimation.

In the second application, the aim is to diagnose 56 women with cervical lesions using 2576 sequenced microRNAs. We include a grouping of the features to enhance predictive performance and feature selection. In this example, gren outperforms the regular elastic net with respect to predictive performance for a range of model sizes. Random forest is competitive to gren, but requires all features.

## 6. Discussion

In a taxonomy of Bayesian methods, gren may be considered a local shrinkage model, as opposed to the global–local shrinkage priors that Polson and Scott (2012) discuss. They characterize certain desirable properties of these global–local shrinkage priors in high dimensions, which, for example, the horseshoe possesses (Carvalho *and others*, 2010). In our case, global shrinkage would imply adding another hyperprior for the global $\lambda_1$ and $\lambda_2$ (or $\alpha$ and $\lambda$) hyperparameters. We argue however, that if the groups are informative, the EB estimation of the (semi-) global shrinkage parameters $\lambda'_g$ may be more beneficial than full Bayes shrinkage of the global penalty parameters, because the latter does not use any known structure to model variability in the hyperparameters. Nonetheless, an interesting direction of future research is the extension of the group-regularized elastic net to a group-regularized horseshoe model, since the horseshoe has been shown to handle sparsity well and render better coverage of credibility intervals than lasso-type priors (van der Pas *and others*, 2014).

Although our method is essentially a reweighted elastic net and can be considered weakly adaptive, it is different from the adaptive lasso (Huang *and others*, 2008; Zou, 2006) and adaptive elastic net (Zou and Zhang, 2009) because it adapts to external information rather than to the primary data. It also differs in the scale of adaptation: in the adaptive lasso and elastic net the adaptive weights are feature specific, while in our case they are estimated on the group level, rendering the adaptation more robust. Both the simulations and the applications illustrate that adaptation to external data may be beneficial for prediction and feature selection for a range of marker types (RNAseqs, microRNAs, and metabolites) due to the ”borrowing of information“ effect: estimates of feature effects that behave similarly are shrunken similarly, yielding overall, better predictions.

As touched upon in Section 1, an obvious comparison is to the group lasso (Meier *and others*, 2008) and its extensions. Although the group lasso is similar in the sense that it shrinks on the group level, it is built upon an entirely different philosophy: its intended application is to small interpretable groups of features, like, for example, gene pathways. Another difference between gren and the group lasso is the form of the penalty: gren estimates one parameter per group. Once these are estimated gren fits a reweighted elastic net. The group lasso uses one overall penalty parameter; it is thereby less flexible in differential shrinkage of the parameters. Our simulations and data applications show that gren is competitive and often superior to (extensions of) the group lasso.

A recent development is to group samples rather than features (Dondelinger and Mukherjee, 2018) to allow different levels of sparsity across sample groups. This approach does not incorporate feature information, and uses CV to estimate the penalty parameters. An interesting future line of research would be to combine this approach with gren.

A common criticism of the lasso (and elastic net) is its instability of feature selection: different data instances lead to different sets of selected features. To investigate the stability of selection on the data from Section 5.2 we created bootstrap samples from the original data and calculated the sizes of the selected feature set intersections. Compared to the regular elastic net, the inclusion of extra information increases the selection stability (see Section 11 of the supplementary material available at *Biostatistics* online). In addition, we investigated penalty multiplier estimation on 100 random partitionings of the features (Section 11 of the supplementary material available at *Biostatistics* online). We found that the estimated penalty parameters tend to cluster around one, as desired with random groups.

A possible weak point of the proposed method is the double EM loop, which may end up in a local optimum, depending on the starting values of the algorithm. Reasonable starting values, e.g. obtained by applying GRridge, could alleviate this issue. By default however, gren simply starts from the elastic net, i.e., penalty multipliers equal to one, and then adapts these. In the applications, we investigated the occurrence of multiple optima, but never encountered them. This does not guarantee that local optima do not occur, but it provides some evidence that local optima are not ubiquitous.

Proper uncertainty quantification in the elastic net is an open problem. If uncertainty quantification is required, Gibbs samples from the posterior (with the estimated hyperparameters) could be drawn. However, we do not recommend Bayesian uncertainty quantification for the Bayesian elastic net due to bad frequentist properties of lasso-like posteriors (Castillo *and others*, 2015) in sparse (omics) settings. Hence, selected features should be interpreted with caution but are nonetheless deemed useful in prediction.

An EM algorithm runs the danger of excessive computation time. In our implementation, we have reduced computational time considerably by some computational shortcuts (see Section 7 of the supplementary material available at *Biostatistics* online) and implementing some parts in C++. To assess computation times we compared gren other methods introduced in Sections 5.1– 5.3 on a Macbook Pro 2016 running $\times$86.64, darwin15.6.0 and present the results in Section 8 of the supplementary material available at *Biostatistics* online. In general, we found that gren is similar in times as GRridge, faster than SGL, and slower than cMCP, and gel.

## Supplementary Material

kxz062_Supplementary_Data

## Software

A (stable) R package is available from https://CRAN.R-project.org/package=gren.

## Reproducible research

All results and documents may be recreated from https://github.com/magnusmunch/gren.
