# Supplementary material for: Adaptive group-regularized logistic elastic net regression
Source: Biostatistics. 2019 Dec 30;22(4):723–37. doi: 10.1093/biostatistics/kxz062 (PMC8596493; doi:10.1093/biostatistics/kxz062)
Supplement: kxz062_Supplementary_Data [file kxz062_supplementary_data.zip › biosts-19093-File046.pdf]

Supplementary material to 'Adaptive group-regularized logistic elastic net regression'

Magnus M. Münch[1,2,*], Carel F.W. Peeters[1], Aad W. van der Vaart[2], and Mark A. van de Wiel[1,3]

December 3, 2019

1. Department of Epidemiology & Biostatistics, Amsterdam Public Health research institute, Amsterdam University medical centers, PO Box 7057, 1007 MB Amsterdam, The Netherlands
2. Mathematical Institute, Leiden University, Leiden, The Netherlands
3. MRC Biostatistics Unit, Cambridge University, Cambridge, United Kingdom

Keywords: Empirical Bayes; High-dimensional data; Prediction; Variational Bayes

Software available from: `https://CRAN.R-project.org/package=gren`

1 Introduction

This document contains supplementary material to the paper 'Adaptive group-regularized logistic elastic net regression'. Section 2 contains extra details on the elastic net prior distribution. Section 3 gives the objective function that is maximised in the variational Bayes procedure introduced in Section 4 of the Main Document (MD). Section 4 gives details on the Pólya-Gamma parametrisation introduced in Section 4 of the MD. The derivations of the variational posterior used in the MD are shown in Section 5. In Section 6 the variational and empirical Bayes updating equations for the algorithm are given. Section 7 shows how we may reduce the computational complexity of our variational Bayes procedure. The computational times of the method are explored in Section 8. Extensions of our method are presented in Section 9. We introduce the ridge and lasso versions of the proposed method in Section 10. Additional results to the applications in the MD Sections 5.2–5.3 are presented in Sections 11–12. We apply our method to a study on metabolomics in Alzheimer's in Section 13 and to study in microRNAs in cervical cancer data in Section 14. Finally, the simulations in MD Section 5.1 are elaborated in Section 15.

2 The elastic net prior

2.1 Density function

The density function of β, drawn from an elastic net prior distribution, is given by:

$$f(\beta) = g(\lambda_1, \lambda_2) \exp\left[-\frac{1}{2}(\lambda_1|\beta| + \lambda_2\beta^2)\right].$$

*Correspondence to: m.munch@vumc.nl

The normalizing constant here is calculated as:

$$g(\lambda_1, \lambda_2) = \left\{ \int_{-\infty}^{\infty} \exp\left[-\frac{1}{2}(\lambda_1|\beta| + \lambda_2\beta^2) \right] d\beta \right\}^{-1} = \frac{\sqrt{\lambda_2}}{2} \phi\left(\frac{\lambda_1}{2\sqrt{\lambda_2}} \right) \Phi\left(\frac{-\lambda_1}{2\sqrt{\lambda_2}} \right)^{-1},$$

with $\phi(x)$ and $\Phi(x)$ the standard normal density and distribution functions of x, respectively.

2.2 Expectation and variance

The expectation and variance of β are:

$$\begin{aligned}
\mathbb{E}[\beta] &= g(\lambda_1, \lambda_2) \int_{-\infty}^{\infty} \beta \cdot \exp\left[-\frac{1}{2}(\lambda_1|\beta| + \lambda_2\beta^2) \right] d\beta = 0, \\
\mathbb{V}[\beta] &= g(\lambda_1, \lambda_2) \int_{-\infty}^{\infty} \beta^2 \cdot \exp\left[-\frac{1}{2}(\lambda_1|\beta| + \lambda_2\beta^2) \right] d\beta - \mathbb{E}[\beta]^2 \\
&= \frac{\lambda_1^2}{4\lambda_2^2} + \frac{1}{\lambda_2} - \frac{\lambda_1}{2\lambda_2^{3/2}} \phi\left(\frac{\lambda_1}{2\sqrt{\lambda_2}} \right) \Phi\left(\frac{-\lambda_1}{2\sqrt{\lambda_2}} \right)^{-1}.
\end{aligned}$$

2.3 Simulation

Generating samples from the elastic net prior is not straightforward. Li and Lin (2010) show that the elastic net prior may be written as a scale mixture of normals, with mixing parameter $\boldsymbol{\tau} = \begin{bmatrix} \tau_1 & \cdots & \tau_p \end{bmatrix}^{\mathrm{T}}$:

$$\begin{aligned}
\beta|\tau &\sim \mathcal{N}\left(0, \frac{1}{\lambda_2} \frac{\tau - 1}{\tau} \right), \\
\tau &\sim \mathcal{TG}\left(\frac{1}{2}, \frac{8\lambda_2}{\lambda_1^2}, (1, \infty) \right),
\end{aligned}$$

where $\mathcal{TG}(k, \theta, (x_l, x_u))$ denotes the truncated gamma distribution with shape k, scale θ, and domain (x_l, x_u). So with an efficient truncated gamma sampler available we may sample from the prior using any standard software package that includes a Gaussian sampling routine. More specifically, we require a a sample from the $\mathcal{TG}\left(\frac{1}{2}, 8\lambda2/\lambda_1^2, (1, \infty)\right)$ distribution. The CDF of this distribution is given by:

$$F_T(\tau) = \begin{cases} 0, & \text{if } \tau < 1 \\ 1 - \dfrac{\Phi\left(-\frac{\lambda_1}{2} \sqrt{\frac{\tau}{\lambda_2}} \right)}{\Phi\left(-\frac{\lambda_1}{2\sqrt{\lambda_2}} \right)}, & \text{if } \tau \geq 1. \end{cases}$$

Since $F_T(\tau)$ is continuous and strictly monotonically increasing for $\tau \geq 1$, the quantile function may be written as a function of the probability $p \in (0, 1)$:

$$Q_T(p) = F_T^{-1}(p) = \frac{4\lambda_2}{\lambda_1^2} \Phi^{-1} \left[(1 - p)\Phi\left(-\frac{\lambda_1}{2\sqrt{\lambda_2}} \right) \right]^2,$$

where $\Phi^{-1}(p)$ is the quantile function of the standard normal distribution, i.e, the probit function. Employing inverse transform sampling for the truncated gamma distribution, an elastic net prior sampling scheme is now:

Generate $U \sim \mathcal{U}(0, 1)$

Set $\tau = \frac{4\lambda_2}{\lambda_1^2}\Phi^{-1}\left[(1-u)\Phi\left(-\frac{\lambda_1}{2\sqrt{\lambda_2}}\right)\right]^2$
Set $\nu^2 = \frac{\tau-1}{\tau\lambda_2}$
Generate $\beta \sim \mathcal{N}(0, \nu^2)$
return β

3 Objective function

The lower bound on the marginal likelihood, or evidence lower bound (ELBO), at iteration t is given by:

$$\begin{aligned}\text{ELBO}(Q^{(t)}) &= \mathbb{E}_{Q^{(t)}}[\log \mathcal{L}(\mathbf{y}, \boldsymbol{\omega}, \boldsymbol{\beta}, \boldsymbol{\tau})] - \mathbb{E}_{Q^{(t)}}[\log Q(\boldsymbol{\omega}, \boldsymbol{\beta}, \boldsymbol{\tau})] && \text{(1a)}\\ &= \mathbb{E}_{Q^{(t)}}[\log \mathcal{L}(\mathbf{y}; \boldsymbol{\beta})] + \mathbb{E}_{Q^{(t)}}[\log \pi(\boldsymbol{\omega}|\boldsymbol{\beta})] - \mathbb{E}_{Q^{(t)}}[\log q(\boldsymbol{\omega})] && \text{(1b)}\\ &+ \mathbb{E}_{Q^{(t)}}[\log \pi(\boldsymbol{\beta}|\boldsymbol{\tau})] - \mathbb{E}_{Q^{(t)}}[\log q(\boldsymbol{\beta})] && \text{(1c)}\\ &+ \mathbb{E}_{Q^{(t)}}[\log \pi(\boldsymbol{\tau})] - \mathbb{E}_{Q^{(t)}}[\log q(\boldsymbol{\tau})]. && \text{(1d)}\end{aligned}$$

We start with the computation of (1b). To this end we define:

$$h(m_i, \omega_i) := \sum_{k=0}^{\infty}(-1)^k \frac{\Gamma(k+m_i}{k+1}\frac{2k+m_i}{\sqrt{2\pi\omega_i^3}}\exp\left[-\frac{(2k+m_i)^2}{8\omega_i}\right].$$

Let $\mathbf{c} = \begin{bmatrix} c_1 & \cdots & c_n \end{bmatrix}^{\mathrm{T}}$. Furthermore, in the following, all expectations and variances are with respect to $Q^{(t)}$. Next we write out the three terms in (1b):

$$\begin{aligned}\mathbb{E}[\log \mathcal{L}(\mathbf{y}; \boldsymbol{\beta})] &= \sum_{i=1}^{n} \log \binom{m_i}{y_i} + \mathbf{y}^{\mathrm{T}}\mathbf{X}\mathbb{E}[\boldsymbol{\beta}] - \mathbf{m}^{\mathrm{T}}\mathbb{E}\left\{\log[1+\exp(\mathbf{x}_i^{\mathrm{T}}\boldsymbol{\beta})]\right\}, && \text{(2a)}\\ \mathbb{E}[\log \pi(\boldsymbol{\omega}|\boldsymbol{\beta})] &= \mathbf{m}^{\mathrm{T}}\mathbb{E}\left\{\log[1+\exp(\mathbf{x}_i^{\mathrm{T}}\boldsymbol{\beta})]\right\} - n\log 2 - \frac{1}{2}\mathbf{m}^{\mathrm{T}}\mathbf{X}\mathbb{E}[\boldsymbol{\beta}] && \text{(2b)}\\ &- \sum_{i=1}^{n}\log\Gamma(m_i) - \frac{1}{2}\sum_{i=1}^{n}[(\mathbf{x}_i^{\mathrm{T}}\mathbb{E}[\boldsymbol{\beta}])^2 + \mathbf{x}_i^{\mathrm{T}}\mathbb{V}[\boldsymbol{\beta}]\mathbf{x}_i]\mathbb{E}[\omega_i] && \text{(2c)}\\ &+ \sum_{i=1}^{n}\mathbb{E}\left\{\log[h(m_i, \omega_i)]\right\}, && \text{(2d)}\\ \mathbb{E}[\log q(\boldsymbol{\omega})] &= \mathbf{m}^{\mathrm{T}}\log[1+\exp(\mathbf{c})] - n\log 2 - \frac{1}{2}\mathbf{m}^{\mathrm{T}}\mathbf{c} - \sum_{i=1}^{n}\log\Gamma(m_i) - \frac{1}{2}\sum_{i=1}^{n}c_i^2\mathbb{E}[\omega_i] && \text{(2e)}\\ &+ \sum_{i=1}^{n}\mathbb{E}\left\{\log[h(m_i, \omega_i)]\right\}, && \text{(2f)}\end{aligned}$$

where all mathematical operations on vectors and matrices are element wise. After combining the terms in (2) and substituting the expectations and variances, several terms cancel to give:

$$\begin{aligned}&\mathbb{E}_{Q^{(t)}}[\log \mathcal{L}(\mathbf{y}; \boldsymbol{\beta})] + \mathbb{E}_{Q^{(t)}}[\log \pi(\boldsymbol{\omega}|\boldsymbol{\beta})] - \mathbb{E}_{Q^{(t)}}[\log q(\boldsymbol{\omega})] && \text{(3a)}\\ &= \sum_{i=1}^{n}\log\binom{m_i}{y_i} + (\mathbf{y} - \frac{1}{2}\mathbf{m})^{\mathrm{T}}\mathbf{X}\boldsymbol{\mu}^{(t)} + \mathbf{m}^{\mathrm{T}}\left\{\frac{1}{2}\mathbf{c}^{(t)} - \log[1+\exp(\mathbf{c}^{(t)})]\right\} && \text{(3b)}\\ &+ \frac{1}{4}\sum_{i=1}^{n}\frac{m_i}{c_i^{(t)}}\tanh\left(\frac{c_i^{(t)}}{2}\right)\left\{(c_i^{(t)})^2 - (\mathbf{x}_i^{\mathrm{T}}\boldsymbol{\mu}^{(t)})^2 - \mathbf{x}_i^{\mathrm{T}}\boldsymbol{\Sigma}^{(t)}\mathbf{x}_i^{\mathrm{T}}\right\}. && \text{(3c)}\end{aligned}$$

Inspection of the updating equations (13), learns us that the last term in the right-hand side of (3) equals zero, so that we are left with just (3b). After a change of variables $\psi_j = \tau_j - 1$, the two terms in (1c) are as follows:

$$
\begin{aligned}
\mathbb{E}[\log \pi(\boldsymbol{\beta}|\boldsymbol{\tau})] = &\frac{1}{2}\sum_{g=1}^{G} |\mathcal{G}(g)| \log \lambda'_g + \frac{p}{2}\log\lambda_2 - \frac{p}{2}\log(2\pi) &\text{(4a)}\\
&- \frac{\lambda_2}{2}\sum_{g=1}^{G}\lambda'_g \sum_{j\in\mathcal{G}(g)} \left(1 + \frac{\lambda_1}{2\sqrt{\lambda_2\chi_j}}\right)(\boldsymbol{\mu}_j^2 + \boldsymbol{\Sigma}_{jj}) &\text{(4b)}\\
&- \frac{1}{2}\sum_{j=1}^{p}\mathbb{E}[\log\psi_j] + \frac{1}{2}\sum_{j=1}^{p}\mathbb{E}[\log(\psi_j+1)], &\text{(4c)}\\
\mathbb{E}[\log q(\boldsymbol{\beta})] = &-\frac{p}{2} - \frac{p}{2}\log(2\pi) - \frac{p}{2}\log|\boldsymbol{\Sigma}|. &\text{(4d)}
\end{aligned}
$$

We combine the terms in (4) and arrive at the following for (1c):

$$
\begin{aligned}
&\mathbb{E}_{Q^{(t)}}[\log \pi(\boldsymbol{\beta}|\boldsymbol{\tau})] - \mathbb{E}_{Q^{(t)}}[\log q(\boldsymbol{\beta})] &\text{(5a)}\\
&= \frac{1}{2}\sum_{g=1}^{G}|\mathcal{G}(g)|\log\lambda_g'^{(t)} + \frac{p}{2}\log\lambda_2 + \frac{p}{2}\log|\boldsymbol{\Sigma}^{(t)}| + \frac{p}{2} &\text{(5b)}\\
&- \frac{\lambda_2}{2}\sum_{g=1}^{G}\lambda_g'^{(t)}\sum_{j\in\mathcal{G}(g)}\left(1 + \frac{\lambda_1}{2\sqrt{\lambda_2\chi_j^{(t)}}}\right)\left((\boldsymbol{\mu}_j^{(t)})^2 + \boldsymbol{\Sigma}_{jj}^{(t)}\right) &\text{(5c)}\\
&- \frac{1}{2}\sum_{j=1}^{p}\mathbb{E}_{Q^{(t)}}[\log\psi_j] + \frac{1}{2}\sum_{j=1}^{p}\mathbb{E}_{Q^{(t)}}[\log(\psi_j+1)]. &\text{(5d)}
\end{aligned}
$$

Repeating the same exercise for (1d) gives:

$$
\begin{aligned}
\mathbb{E}[\log\pi(\boldsymbol{\tau})] = &\, p\log\lambda_1 - \frac{p}{2}\log\lambda_2 - \frac{5p}{2}\log 2 - \frac{p}{2}\log\pi - \frac{p}{2} - \frac{p\lambda_1^2}{8\lambda_2} &\text{(6a)}\\
&- p\log\Phi\left(\frac{-\lambda_1}{2\sqrt{\lambda_2}}\right) - \frac{\lambda_1}{4\sqrt{\lambda_2}}\sum_{j=1}^{p}\chi_j^{1/2} - \frac{1}{2}\sum_{j=1}^{p}\mathbb{E}[\log(\psi_j+1)], &\text{(6b)}\\
\mathbb{E}[\log q(\boldsymbol{\tau})] = &\, p\log\lambda_1 - \frac{p}{2}\log\lambda_2 - \frac{3p}{2}\log 2 - \frac{p}{2}\log\pi - \frac{p}{2} - \frac{\lambda_1}{2\sqrt{\lambda_2}}\sum_{j=1}^{p}\chi_j^{1/2} &\text{(6c)}\\
&+ \frac{1}{4}\sum_{j=1}^{p}\log\chi_j - \frac{1}{2}\sum_{j=1}^{p}\mathbb{E}[\log\psi_j]. &\text{(6d)}
\end{aligned}
$$

Again, we combine the terms in (6) to get:

$$
\begin{aligned}
&\mathbb{E}_{Q^{(t)}}[\log\pi(\boldsymbol{\tau})] - \mathbb{E}_{Q^{(t)}}[\log q(\boldsymbol{\tau})] &\text{(7a)}\\
&= p\log 2 - \frac{p\lambda_1^2}{8\lambda_2} - p\log\Phi\left(\frac{-\lambda_1}{2\sqrt{\lambda_2}}\right) + \frac{\lambda_1}{4\sqrt{\lambda_2}}\sum_{j=1}^{p}\sqrt{\chi_j^{(t)}} - \frac{1}{4}\sum_{j=1}^{p}\log\chi_j^{(t)} &\text{(7b)}\\
&+ \frac{1}{2}\sum_{j=1}^{p}\mathbb{E}_{Q^{(t)}}[\log\psi_j] - \frac{1}{2}\sum_{j=1}^{p}\mathbb{E}_{Q^{(t)}}[\log(\psi_j+1)]. &\text{(7c)}
\end{aligned}
$$

Adding up the terms in (3), (5), and (7) gives the ELBO, at iteration t:

$$\begin{aligned}\text{ELBO}(Q^{(t)}) \propto\ & (\mathbf{y} - \frac{1}{2}\mathbf{m})^{\mathrm{T}}\mathbf{X}\boldsymbol{\mu}^{(t)} + \mathbf{m}^{\mathrm{T}}\{\frac{1}{2}\mathbf{c}^{(t)} - \log[\exp(\mathbf{c}^{(t)}) + 1]\} + \frac{1}{2}\sum_{g=1}^{G} |\mathcal{G}(g)| \log \lambda_g'^{(t)} \\ & + \frac{1}{2}\log|\boldsymbol{\Sigma}^{(t)}| - \frac{\lambda_2}{2}\sum_{g=1}^{G}\lambda_g'^{(t)} \sum_{j\in\mathcal{G}(g)} \left(1 + \frac{\lambda_1}{2\sqrt{\lambda_2\chi_j^{(t)}}}\right)\left((\boldsymbol{\mu}_j^{(t)})^2 + \boldsymbol{\Sigma}_{jj}^{(t)}\right) \\ & + \frac{\lambda_1}{4\sqrt{\lambda_2}}\sum_{j=1}^{p}\sqrt{\chi_j^{(t)}} - \frac{1}{4}\sum_{j=1}^{p}\log\chi_j^{(t)}.\end{aligned}$$

Here, proportionality is with respect to the variational and penalty parameters.

4 The Pólya-Gamma parametrisation

In Section 4 of the MD latent Pólya-Gamma distributed variables are introduced. Likewise, in Polson et al. (2013), Pólya-Gamma variables are introduced into the logistic regression model under a Gaussian prior. Here, we explain how introduction of these latent variables simplifies the variational Bayes calculations.

In Polson et al. (2013), the central identity for this parametrisation is given in their Theorem 1:

$$\frac{\exp(\psi)^a}{[1+\exp(\psi)]^b} = 2^{-b}\exp(\kappa\psi)\int_0^\infty \exp(-\omega\psi^2/2)\pi(\omega)d\omega, \tag{8}$$

where $\kappa = a - b/2$ and ω is $\mathcal{PG}\,(b, 0)$ distributed. This holds for all $b > 0$ and $a \in \mathbb{R}$. After setting $a = y_i$, $b = m_i$, and $\psi = \mathbf{x}_i^{\mathrm{T}}\boldsymbol{\beta}$, we may consider the right-hand side of (8) as an un-normalised joint density in $(\boldsymbol{\beta}, \omega)$. The likelihood contribution of observation i for $\boldsymbol{\beta}$ conditional on ω_i may now be written as:

$$\mathcal{L}_i(\boldsymbol{\beta}|\omega_i) \propto \exp[\kappa_i\mathbf{x}_i^{\mathrm{T}}\boldsymbol{\beta} - \omega_i(\mathbf{x}_i^{\mathrm{T}}\boldsymbol{\beta})^2/2].$$

This allows the conditional posterior of $\boldsymbol{\beta}$ to be written as:

$$p(\boldsymbol{\beta}|\boldsymbol{\omega},\mathbf{y}) \propto \pi(\boldsymbol{\beta})\prod_{i=1}^{n}\mathcal{L}_i(\boldsymbol{\beta}|\omega_i) = \pi(\boldsymbol{\beta})\exp\left[-\frac{1}{2}(\boldsymbol{\Omega}^{-1}\boldsymbol{\kappa} - \mathbf{X}\boldsymbol{\beta})^{\mathrm{T}}\boldsymbol{\Omega}(\boldsymbol{\Omega}^{-1}\boldsymbol{\kappa} - \mathbf{X}\boldsymbol{\beta})\right],$$

where $\boldsymbol{\Omega} = \text{diag}(\omega_i)$, for $i = 1, \dots, n$. Consequently, with $\pi(\boldsymbol{\beta})$ a Gaussian prior, $p(\boldsymbol{\beta}|\boldsymbol{\omega},\mathbf{y})$ is also in the Gaussian family.

Furthermore, by treating the integrand in (8) as an un-normalized density over (ψ, ϕ), we find the distribution of ω conditional on $\boldsymbol{\beta}$,

$$p(\omega|\psi) = \frac{\exp(-\omega\psi^2/2)\pi(\omega)}{\int_0^\infty \exp(-\omega\psi^2/2)\pi(\omega)d\omega},$$

distributed as $\omega|\psi \sim \mathcal{PG}(b,\psi)$ (Polson et al., 2013), with expectation $\mathbb{E}[\omega] = \frac{b}{2\psi}\tanh(\psi/2)$. We substitute again $b = m_i$ and $\psi = \mathbf{x}_i^{\mathrm{T}}\boldsymbol{\beta}$, and set the prior $\pi(\boldsymbol{\beta})$ to a Gaussian with mean $\mathbf{m}$ and covariance $\mathbf{V}$ to arrive at the following conditional posteriors:

$$\omega_i|\boldsymbol{\beta} \sim \mathcal{PG}(m_i, \mathbf{x}_i^{\mathrm{T}}\boldsymbol{\beta}) \tag{9a}$$

$$\boldsymbol{\beta}|\mathbf{y},\boldsymbol{\omega} \sim \text{N}(\boldsymbol{\mu},\boldsymbol{\Sigma}), \tag{9b}$$

where

$$\boldsymbol{\Sigma} = (\mathbf{X}^{\mathrm{T}}\boldsymbol{\Omega}\mathbf{X} + \mathbf{V}^{-1})^{-1},$$
$$\boldsymbol{\mu} = \mathbf{V}(\mathbf{X}^{\mathrm{T}}\boldsymbol{\kappa} + \mathbf{V}^{-1}\mathbf{m}),$$

with $\boldsymbol{\kappa} = \begin{bmatrix}\kappa_1 \dots \kappa_n\end{bmatrix}^{\mathrm{T}}$. Equations (9) constitute the Gibbs sampler used in Polson et al. (2013). However, from the corresponding variational Bayes derivations in Section 5, it is obvious that the parametrisation above is also useful in the variational Bayes approximation to the posterior distribution.

5 Variational posterior

The optimal distributions in our variational Bayes implementation are:

$$q^*_{\boldsymbol{\beta}}(\boldsymbol{\beta}) \propto \exp\{\mathbb{E}_{\boldsymbol{\omega},\boldsymbol{\tau}}[\log p(\boldsymbol{\beta},\boldsymbol{\omega},\boldsymbol{\tau},\mathbf{y})]\} \propto \exp\{\log \mathcal{L}(\mathbf{y};\boldsymbol{\beta}) + \mathbb{E}_{\boldsymbol{\omega}}[\log \pi(\boldsymbol{\omega}|\boldsymbol{\beta})] + \mathbb{E}_{\boldsymbol{\tau}}[\log \pi(\boldsymbol{\beta}|\boldsymbol{\tau})]\}, \quad (10)$$
$$q^*_{\boldsymbol{\omega}}(\boldsymbol{\omega}) \propto \exp\{\mathbb{E}_{\boldsymbol{\beta},\boldsymbol{\tau}}[\log p(\boldsymbol{\beta},\boldsymbol{\omega},\boldsymbol{\tau},\mathbf{y})]\} \propto \exp\{\mathbb{E}_{\boldsymbol{\beta}}[\log \pi(\boldsymbol{\omega}|\boldsymbol{\beta})]\}, \quad (11)$$
$$q^*_{\boldsymbol{\tau}}(\boldsymbol{\tau}) \propto \exp\{\mathbb{E}_{\boldsymbol{\beta},\boldsymbol{\omega}}[\log p(\boldsymbol{\beta},\boldsymbol{\omega},\boldsymbol{\tau},\mathbf{y})]\} \propto \exp\{\mathbb{E}_{\boldsymbol{\beta}}[\log \pi(\boldsymbol{\beta}|\boldsymbol{\tau})] + \log \pi(\boldsymbol{\tau})\}. \quad (12)$$

For convenience we work on the log-scale and occasionally do a change of variables $\psi_j = \tau_j - 1$. Starting with $\boldsymbol{\beta}$ we have for the three terms on the right-hand side of (10):

$$\begin{aligned}
\log \mathcal{L}(\mathbf{y};\boldsymbol{\beta}) &= \sum_{i=1}^{n} \log f(y_i;\boldsymbol{\beta}) \propto \sum_{i=1}^{n} \log \left\{ \frac{\exp(\mathbf{x}_i^{\mathrm{T}}\boldsymbol{\beta})^{y_i}}{[1+\exp(\mathbf{x}_i^{\mathrm{T}}\boldsymbol{\beta})]^{m_i}} \right\} \\
&= \mathbf{y}^{\mathrm{T}}\mathbf{X}\boldsymbol{\beta} - \sum_{i=1}^{n} m_i \log[1+\exp(\mathbf{x}_i^{\mathrm{T}}\boldsymbol{\beta})], \\
\mathbb{E}_{\boldsymbol{\omega}}[\log \pi(\boldsymbol{\omega}|\boldsymbol{\beta})] &\propto \sum_{i=1}^{n} \mathbb{E}_{\omega_i}[\log \pi(\omega_i|\boldsymbol{\beta})] \\
&\propto \sum_{i=1}^{n} \mathbb{E}_{\omega_i}\left(\log\left\{ \cosh\left(\frac{\mathbf{x}_i^{\mathrm{T}}\boldsymbol{\beta}}{2}\right)^{m_i} \exp\left[-\frac{(\mathbf{x}_i^{\mathrm{T}}\boldsymbol{\beta})^2}{2}\omega_i\right]\right\}\right) \\
&\propto \sum_{i=1}^{n} m_i \log\cosh\left(\frac{\mathbf{x}_i^{\mathrm{T}}\boldsymbol{\beta}}{2}\right) - \frac{1}{2}\boldsymbol{\beta}^{\mathrm{T}}\mathbf{X}^{\mathrm{T}}\boldsymbol{\Omega}\mathbf{X}\boldsymbol{\beta} \\
&\propto \sum_{i=1}^{n} m_i \log[1+\exp(\mathbf{x}_i^{\mathrm{T}}\boldsymbol{\beta})] - \frac{1}{2}\mathbf{m}^{\mathrm{T}}\mathbf{X}\boldsymbol{\beta} - \frac{1}{2}\boldsymbol{\beta}^{\mathrm{T}}\mathbf{X}^{\mathrm{T}}\boldsymbol{\Omega}\mathbf{X}\boldsymbol{\beta}, \\
\mathbb{E}_{\boldsymbol{\tau}}[\log \pi(\boldsymbol{\beta}|\boldsymbol{\tau})] &= \sum_{j=1}^{p} \mathbb{E}_{\tau_j}[\log \pi(\beta_j|\tau_j)] \propto \frac{\lambda_2}{2} \sum_{j=1}^{p} \beta_j^2 \lambda'_{g(j)} \mathbb{E}\left(\frac{\tau_j}{\tau_j - 1}\right) \\
&= \sum_{j=1}^{p} \beta_j^2 \lambda'_{g(j)} [1 + \mathbb{E}(\psi_j^{-1})] = -\frac{1}{2}\lambda_2 \boldsymbol{\beta}^{\mathrm{T}}\boldsymbol{\Lambda}'(\mathbf{I}+\mathbf{Z})\boldsymbol{\beta},
\end{aligned}$$

where proportionality is with respect to $\boldsymbol{\beta}$. Furthermore, $\boldsymbol{\Omega} = \mathrm{diag}[\mathbb{E}(\omega_i)]$, $\mathbf{Z} = \mathrm{diag}[\mathbb{E}(\psi_j^{-1})]$, and $\boldsymbol{\Lambda}' = \mathrm{diag}(\lambda'_{g(j)})$. Since the difficult $\sum_{i=1}^{n} m_i \log[1+\exp(\mathbf{x}_i^{\mathrm{T}}\boldsymbol{\beta})]$ term appears in both the $\log \mathcal{L}(\mathbf{y};\boldsymbol{\beta})$ and $\pi(\omega_i|\boldsymbol{\beta})$ part, it cancels, thereby justifying the introduction of the ω_i. Finally, we have:

$$\log q^*_{\boldsymbol{\beta}}(\boldsymbol{\beta}) = \boldsymbol{\kappa}^{\mathrm{T}}\mathbf{X}\boldsymbol{\beta} - \frac{1}{2}\boldsymbol{\beta}^{\mathrm{T}}(\mathbf{X}^{\mathrm{T}}\boldsymbol{\Omega}\mathbf{X} + \lambda_2\boldsymbol{\Lambda}' + \lambda_2\boldsymbol{\Lambda}'\mathbf{Z})\boldsymbol{\beta},$$

which is quadratic in $\boldsymbol{\beta}$ and thus recognised as the log-kernel of a Gaussian distribution with covariance $\boldsymbol{\Sigma} = (\mathbf{X}^{\mathrm{T}}\boldsymbol{\Omega}\mathbf{X} + \lambda_2\boldsymbol{\Lambda}' + \lambda_2\boldsymbol{\Lambda}'\mathbf{Z})^{-1}$ and mean $\boldsymbol{\mu} = \boldsymbol{\Sigma}\mathbf{X}^{\mathrm{T}}\boldsymbol{\kappa}$.

Next we derive the variational posterior for $\boldsymbol{\omega}$ in (11):

$$\begin{aligned}
\log q^*_{\boldsymbol{\omega}}(\boldsymbol{\omega}) &= \mathbb{E}_{\boldsymbol{\beta}}[\log \pi(\boldsymbol{\omega}|\boldsymbol{\beta})] = \sum_{i=1}^{n} \mathbb{E}_{\boldsymbol{\beta}}[\log \pi(\omega_i|\boldsymbol{\beta})] \\
&= \sum_{i=1}^{n} \mathbb{E}_{\boldsymbol{\beta}} \left(m_i \log \left\{ \cosh\left(\frac{\mathbf{x}_i^{\mathrm{T}}\boldsymbol{\beta}}{2}\right) \exp\left[-\frac{(\mathbf{x}_i^{\mathrm{T}}\boldsymbol{\beta})^2}{2}\omega_i\right] \cdot f(\omega_i|m_i, 0)\right\}\right) \\
&= \sum_{i=1}^{n} \mathbb{E}_{\boldsymbol{\beta}} \left[m_i \log\cosh\left(\frac{\mathbf{x}_i^{\mathrm{T}}\boldsymbol{\beta}}{2}\right)\right] - \frac{1}{2}\sum_{i=1}^{n} \omega_i \mathbb{E}[(\mathbf{x}_i^{\mathrm{T}}\boldsymbol{\beta})^2] + \sum_{i=1}^{n} \log f(\omega_i|m_i, 0) \\
&\propto \sum_{i=1}^{n} m_i \log\cosh\left\{\frac{\sqrt{[\mathbf{x}_i^{\mathrm{T}}\mathbb{E}(\boldsymbol{\beta})]^2 + \mathbf{x}_i^{\mathrm{T}}\mathbb{V}[\boldsymbol{\beta}]}}{2}\right\} - \frac{1}{2}\sum_{i=1}^{n} \omega_i \left\{[\mathbf{x}_i^{\mathrm{T}}\mathbb{E}(\boldsymbol{\beta})]^2 + \mathbf{x}_i^{\mathrm{T}}\mathbb{V}[\boldsymbol{\beta}]\right\} \\
&\quad + \sum_{i=1}^{n} \log f(\omega_i|m_i, 0),
\end{aligned}$$

where proportionality is with respect to $\boldsymbol{\omega}$. If exponentiated, this is a product of tilted and renormalised $\mathcal{PG}(b_i, c_i)$ variables (Polson et al., 2013), with $b_i = m_i$ and $c_i = \sqrt{[\mathbf{x}_i^{\mathrm{T}}\mathbb{E}(\boldsymbol{\beta})]^2 + \mathbf{x}_i^{\mathrm{T}}\mathbb{V}[\boldsymbol{\beta}]\mathbf{x}_i}$.

The variational posterior terms of $\boldsymbol{\tau}$ in (12) are calculated as:

$$\begin{aligned}
\mathbb{E}_{\boldsymbol{\beta}}[\log \pi(\boldsymbol{\beta}|\boldsymbol{\tau})] &= \sum_{j=1}^{p} \mathbb{E}_{\beta_j}[\log \pi(\beta_j|\tau_j)] \propto \frac{1}{2}\sum_{j=1}^{p} \log\left(\frac{\tau_j}{\tau_j - 1}\right) - \frac{1}{2}\sum_{j=1}^{p} \mathbb{E}(\beta_j^2)\lambda_2\lambda'_{g(j)}\frac{\tau_j}{\tau_j - 1}, \\
\log \pi(\boldsymbol{\tau}) &= \sum_{j=1}^{p} \log \pi(\tau_j) \propto -\frac{1}{2}\sum_{j=1}^{p} \log \tau_j - \sum_{j=1}^{p} \frac{\lambda_1^2}{8\lambda_2}\tau_j.
\end{aligned}$$

Combining the terms we find the log-kernel of the variational posterior as:

$$\begin{aligned}
\log q^*_{\tau}(\boldsymbol{\tau}) &\propto \frac{1}{2}\sum_{j=1}^{p} \log\left(\frac{\tau_j}{\tau_j - 1}\right) - \frac{1}{2}\sum_{j=1}^{p} \mathbb{E}(\beta_j^2)\lambda_2\lambda'_{g(j)}\frac{\tau_j}{\tau_j - 1} - \frac{1}{2}\sum_{j=1}^{p} \log \tau_j - \sum_{j=1}^{p} \frac{\lambda_1^2}{8\lambda_2}\tau_j \\
&= \frac{1}{2}\sum_{j=1}^{p} \log[(\tau_j - 1)^{-1}] - \frac{1}{2}\sum_{j=1}^{p}\left(\frac{\lambda_1^2}{4\lambda_2}\tau_j + [\mathbb{E}(\beta_j)^2 + \mathbb{V}(\beta_j)]\lambda_2\lambda'_{g(j)}\frac{\tau_j}{\tau_j - 1}\right),
\end{aligned}$$

where proportionality is with respect to $\boldsymbol{\tau}$. Again applying the change of variables $\psi_j = \tau_j - 1$, we have:

$$\log q^*_{\psi}(\psi) \propto \frac{1}{2}\sum_{j=1}^{p} \log \psi_j^{-1} - \frac{1}{2}\sum_{j=1}^{p}\left(\frac{\lambda_1^2}{4\lambda_2}\psi_j + [\mathbb{E}(\beta_j)^2 + \mathbb{V}(\beta_j)]\lambda_2\lambda'_{g(j)}\psi_j^{-1}\right),$$

which is the log-kernel of a $\mathrm{GIG}(1/2, \lambda_1^2/(4\lambda_2), \chi_j)$ random variable with $\chi_j = \lambda_2\lambda'_{g(j)}[\mathbb{E}(\beta_j)^2 + \mathbb{V}(\beta_j)]$.

6 Updating equations

If we fill in the expectations and variances in the variational posterior from Section 5 and maximise with respect to the variational parameters, we arrive at the following updating equations:

$$\mathbf{\Sigma}^{(t+1)} = \left(\mathbf{X}^{\mathrm{T}} \mathbf{\Omega}^{(t)} \mathbf{X} + \lambda_2 \mathbf{\Lambda}' + \frac{\lambda_1 \sqrt{\lambda_2}}{2} \mathbf{\Lambda}' \mathbf{Z}^{(t)} \right)^{-1}, \tag{13a}$$

$$\text{with } \mathbf{\Omega}^{(t)} = \text{diag}\left[\left(\frac{m_i}{2c_i^{(t)}} \right) \tanh \left(\frac{c_i^{(t)}}{2} \right) \right] \text{ and } \mathbf{Z}^{(t)} = \text{diag}\left[(\chi_j^{(t)})^{-1/2} \right],$$

$$\boldsymbol{\mu}^{(t+1)} = \mathbf{\Sigma}^{(t+1)} \mathbf{X}^{\mathrm{T}} (\mathbf{y} - \mathbf{m}/2), \tag{13b}$$

$$c_i^{(t+1)} = \sqrt{\mathbf{x}_i^{\mathrm{T}} \mathbf{\Sigma}^{(t+1)} \mathbf{x}_i + (\mathbf{x}_i^{\mathrm{T}} \boldsymbol{\mu}^{(t+1)})^2}, \text{ for } i = 1, \dots, n, \tag{13c}$$

$$\chi_j^{(t+1)} = \lambda'_{g(j)} \lambda_2 \left[\mathbf{\Sigma}_{jj}^{(t+1)} + (\boldsymbol{\mu}_j^{(t+1)})^2 \right], \text{ for } j = 1, \dots, p, \tag{13d}$$

which we iterate until convergence. Here, $\mathbf{\Lambda}'$ is a diagonal matrix with entries λ'_g, each repeated $|\mathcal{G}(g)|$ times and $\mathbf{m} = \begin{bmatrix} m_1 & \cdots & m_n \end{bmatrix}^{\mathrm{T}}$. Furthermore, $\mathbf{A}_{jj}$ and $\mathbf{a}_j$ denote the jth diagonal element of a square matrix and jth element of a column vector, respectively.

To obtain the empirical Bayes updating equations, we first derive the expected log likelihood under the variational posterior as:

$$\begin{aligned}
\mathbb{E}_Q[\log \mathcal{L}_{\boldsymbol{\lambda}'}(\mathbf{y}, \boldsymbol{\omega}, \boldsymbol{\beta}, \boldsymbol{\tau}) | \boldsymbol{\lambda}'^{(k)}] &\propto \mathbb{E}_Q[\log \pi_{\boldsymbol{\lambda}'}(\boldsymbol{\beta}|\boldsymbol{\tau}) | \boldsymbol{\lambda}'^{(k)}] \propto \sum_{g=1}^{G} \sum_{j \in \mathcal{G}(g)} \mathbb{E}_Q[\log \pi_{\boldsymbol{\lambda}'}(\beta_j|\tau_j) | \boldsymbol{\lambda}'^{(k)}] \\
&\propto \frac{1}{2} \sum_{g=1}^{G} \sum_{j \in \mathcal{G}(g)} \log \lambda'_g - \frac{(1-\alpha)\lambda}{4} \sum_{g=1}^{G} \sum_{j \in \mathcal{G}(g)} \lambda'_g \mathbb{E}_Q \left(\frac{\tau_j}{\tau_j - 1} \beta_j^2 | \boldsymbol{\lambda}'^{(k)} \right) \\
&\propto \frac{1}{2} \sum_{g=1}^{G} |\mathcal{G}(g)| \log \lambda'_g - \frac{(1-\alpha)\lambda}{4} \sum_{g=1}^{G} \lambda'_g d_g^{(k)},
\end{aligned}$$

with the coefficients $d_g^{(k)}$ calculated as:

$$\begin{aligned}
d_g^{(k)} &= \sum_{j \in \mathcal{G}(g)} \mathbb{E}_Q \left(\frac{\tau_j}{\tau_j - 1} \beta_j^2 | \boldsymbol{\lambda}'^{(k)} \right) \\
&= \sum_{j \in \mathcal{G}(g)} \left[1 + \mathbb{E}_{q_{\psi_j}}(\psi_j^{-1} | \boldsymbol{\lambda}'^{(k)}) \right] \left[\mathbb{E}_{q_{\beta_j}}(\beta_j | \boldsymbol{\lambda}'^{(k)})^2 + \mathbb{V}_{q_{\beta_j}}(\beta_j | \boldsymbol{\lambda}'^{(k)}) \right] \\
&= \sum_{j \in \mathcal{G}(g)} \left[\mathbf{\Sigma}_{jj}^{(k)} + (\boldsymbol{\mu}_j^{(k)})^2 \right] \left(1 + \alpha \lambda^{1.5} \sqrt{\frac{1-\alpha}{8\chi_j^{(k)}}} \right).
\end{aligned}$$

Here, $\propto$ denotes proportionality with respect to $\boldsymbol{\lambda}'$. The resulting updating equations for the hyperparameters are now:

$$\boldsymbol{\lambda}'^{(k+1)} = \underset{\boldsymbol{\lambda}'}{\text{argmax}} \frac{1}{2} \sum_{g=1}^{G} |\mathcal{G}(g)| \log(\lambda'_g) - \frac{(1-\alpha)\lambda}{4} \sum_{g=1}^{G} \lambda'_g d_g^{(k)} \tag{14a}$$

$$\text{subject to } \prod_{g=1}^{G} (\lambda'_g)^{|\mathcal{G}(g)|} = 1. \tag{14b}$$

7 Computational complexity

Calculation of $\mathbf{\Sigma}$ involves the inversion of a $(p \times p)$-dimensional matrix, which occurs at every iteration of the algorithm. If naively done, every inversion is of computational complexity $\mathcal{O}(p^3)$. With p large, this is a serious computational bottleneck of the algorithm, especially with possibly many iterations. However, inspection of the updating equations in (13), reveals that we only require the inner products $\mathbf{x}_i^{\mathrm{T}} \mathbf{\Sigma} \mathbf{x}_i$, $\mathbf{x}_i^{\mathrm{T}} \boldsymbol{\mu}$, and $\boldsymbol{\mu}$, plus the diagonal of $\mathbf{\Sigma}$ to directly update the c_i and χ_j. In Section 4 of the MD we show that for the EM updates of $\boldsymbol{\lambda}'$, we only require the c_i, the χ_j, $\boldsymbol{\mu}$, and the diagonal of $\mathbf{\Sigma}$. Here we provide details on how to compute these with complexity $\mathcal{O}(n^2 p)$, thereby considerably reducing the computational cost of the algorithm. If necessary, we may calculate the full $\mathbf{\Sigma}$ once after the algorithm has converged.

In the following we slightly change notation and let $\text{diag}(\mathbf{A})$, with $\mathbf{A}$ a $p \times p$ square matrix, be the operator that extracts the diagonal of $\mathbf{A}$ to a p-dimensional column vector, e.g., $\text{diag}(\mathbf{A}) = \begin{bmatrix} \mathbf{A}_{11} & \cdots & \mathbf{A}_{pp} \end{bmatrix}^{\mathrm{T}}$. We start by calculating the complexity of $\text{diag}(\mathbf{\Sigma})$. To this end, let $\mathbf{H} = \lambda_2 \mathbf{\Lambda}' + \lambda_2 \mathbf{\Lambda}' \mathbf{Z}$. Now, by the Woodbury identity, we may decompose $\text{diag}(\mathbf{\Sigma})$ as:

$$\begin{aligned} \text{diag}(\mathbf{\Sigma}) &= \text{diag}(\mathbf{H}^{-1}) - \text{diag}[\mathbf{H}^{-1}\mathbf{X}^{\mathrm{T}}\mathbf{\Omega}(\mathbf{I} + \mathbf{X}\mathbf{H}^{-1}\mathbf{X}^{\mathrm{T}}\mathbf{\Omega})^{-1}\mathbf{X}\mathbf{H}^{-1}] && \text{(15a)} \\ &= \text{diag}(\mathbf{H}^{-1}) - [(\mathbf{H}^{-1}\mathbf{X}^{\mathrm{T}}\mathbf{\Omega}(\mathbf{I} + \mathbf{X}\mathbf{H}^{-1}\mathbf{X}^{\mathrm{T}}\mathbf{\Omega})^{-1}) \circ \mathbf{H}^{-1}\mathbf{X}^{\mathrm{T}}] \cdot \mathbf{1}_{n\times 1}, && \text{(15b)} \end{aligned}$$

where $\circ$ denotes the Hadamard matrix product and $\mathbf{1}_{p\times n}$ is a $p \times n$-dimensional matrix of ones. By recognising that $\mathbf{H}$ is a diagonal matrix, we can post-multiply it by a $p \times n$-dimensional matrix with computational complexity $\mathcal{O}(np)$, which leads to a total complexity of $\mathcal{O}(n^2 p)$ for $\text{diag}(\mathbf{\Sigma})$. Next we consider the calculation of $\boldsymbol{\mu}$. Again using the Woodbury identity, we may write $\boldsymbol{\mu}$ as:

$$\boldsymbol{\mu} = \mathbf{\Sigma}\mathbf{X}^{\mathrm{T}}\boldsymbol{\kappa} = [\mathbf{H}^{-1}\mathbf{X}^{\mathrm{T}} - \mathbf{H}^{-1}\mathbf{X}^{\mathrm{T}}\mathbf{\Omega}(\mathbf{I} + \mathbf{X}\mathbf{H}^{-1}\mathbf{X}^{\mathrm{T}}\mathbf{\Omega})^{-1}\mathbf{X}\mathbf{H}^{-1}\mathbf{X}^{\mathrm{T}}]\boldsymbol{\kappa}, \quad (16)$$

which is, by a similar argument as before, again an operation of complexity $\mathcal{O}(n^2 p)$.

Now we rewrite the calculation of $\mathbf{c} = \begin{bmatrix} c_1 & \cdots & c_n \end{bmatrix}^{\mathrm{T}}$ as:

$$\begin{aligned} \mathbf{c} &= \begin{bmatrix} \sqrt{\mathbf{x}_1 \mathbf{\Sigma} \mathbf{x}_1 + (\mathbf{x}_1^{\mathrm{T}}\boldsymbol{\mu})^2} & \cdots & \sqrt{\mathbf{x}_n \mathbf{\Sigma} \mathbf{x}_n + (\mathbf{x}_n^{\mathrm{T}}\boldsymbol{\mu})^2} \end{bmatrix}^{\mathrm{T}} \\ &= \sqrt{\text{diag}\left[\mathbf{X}\mathbf{\Sigma}\mathbf{X}^{\mathrm{T}}\right] + \text{diag}\left[\mathbf{X}\boldsymbol{\mu}\boldsymbol{\mu}^{\mathrm{T}}\mathbf{X}^{\mathrm{T}}\right]} \\ &= \sqrt{[(\mathbf{X}\mathbf{\Sigma}) \circ \mathbf{X}] \cdot \mathbf{1}_{p\times 1} + (\mathbf{X}\boldsymbol{\mu}) \circ (\mathbf{X}\boldsymbol{\mu})}. \end{aligned}$$

The calculation requires matrix products of complexity $\mathcal{O}(n^2 p)$, Hadamard products of $\mathcal{O}(np)$ and matrix inversions of $\mathcal{O}(n^3)$, such that the total complexity is of $\mathcal{O}(n^2 p)$.

The parameters $\boldsymbol{\chi} = \begin{bmatrix} \chi_1 & \cdots & \chi_p \end{bmatrix}^{\mathrm{T}}$ are rewritten as:

$$\begin{aligned} \boldsymbol{\chi} &= \begin{bmatrix} \lambda'_{g(1)}\lambda_2(\mathbf{\Sigma}_{11} + \boldsymbol{\mu}_1^2) & \cdots & \lambda'_{g(p)}\lambda_2(\mathbf{\Sigma}_{pp} + \boldsymbol{\mu}_p^2) \end{bmatrix}^{\mathrm{T}} && \text{(17a)} \\ &= \lambda_2 \mathbf{\Lambda}' \left[\text{diag}(\mathbf{\Sigma}) + \text{diag}(\boldsymbol{\mu}\boldsymbol{\mu}^{\mathrm{T}})\right] && \text{(17b)} \\ &= \lambda_2 \boldsymbol{\lambda}' \circ \left[\text{diag}(\mathbf{\Sigma}) + \boldsymbol{\mu} \circ \boldsymbol{\mu}\right]. && \text{(17c)} \end{aligned}$$

By inserting (15) and (16) into (17) we see that the computation of $\boldsymbol{\chi}$ consists of one matrix diagonal of $\mathcal{O}(n^2 p)$, matrix products of complexity $\mathcal{O}(n^2 p)$ and two Hadamard products of $\mathcal{O}(p)$. Computation of $\boldsymbol{\chi}$ is therefore of complexity of $\mathcal{O}(n^2 p)$.

8 Computational times

On the colorectal cancer example in MD Section 5.2 we achieved the following times: 48.81, 38.44, 46.82 seconds, with $\alpha \in \{0.05, 0.5, 0.95\}$, respectively. `GRridge` and `SGL` with $\alpha \in \{0.05, 0.5, 0.95\}$ are slightly faster with 19.46 and 150.97, 146.74, 165.03 seconds, respectively. `cMCP` (3.27, 3.97, 2.92 seconds) and `gel` (3.34, 0.34, 0.02 seconds) are faster than `gren`. The results for the oral cancer example in MD Section 5.3 are similar. `gren` achieved 84.58, 68.37, 83.55 seconds, with $\alpha \in \{0.05, 0.5, 0.95\}$, respectively. `GRridge` was slightly faster with 39.72 seconds, while `SGL` was slightly slower: 429.18, 462.94, 578.21 seconds, with $\alpha \in \{0.05, 0.5, 0.95\}$. `cMCP` (10.07, 8.99, 15.51 seconds) and `gel` (8.82, 12.2, 0.16 seconds) are again faster than `gren`.

9 Extensions

9.1 Unpenalized covariates

Inclusion of an intercept β_0 is achieved by appending the data matrix $\mathbf{X}$ with a column of ones. Penalization of such an intercept parameter is not desirable (Hastie et al., 2009). Additionally, it is often desirable to include unpenalized covariates in the model. Common examples of such covariates in clinical research are patient characteristics, such as age, BMI, and sex. In the following we assume the intercept to be included in the unpenalized covariates.

To include unpenalized covariates, we divide the model matrix into two parts $\mathbf{X} = \begin{bmatrix}\mathbf{X}_u & \mathbf{X}_r\end{bmatrix}$, where $\mathbf{X}_u$ are the u unpenalized variables and $\mathbf{X}_r$ are the r penalized variables. Let $\mathbf{\Lambda}'_*$ and $\mathbf{Z}_*$ be the matrices $\mathbf{\Lambda}'$ and $\mathbf{Z}$ prepended with u zero columns and u zero rows. Then we have for the current estimate of the covariance matrix $\mathbf{\Sigma}$:

$$\begin{aligned}\mathbf{\Sigma} &= \left(\mathbf{X}^{\mathrm{T}}\mathbf{\Omega}\mathbf{X} + \lambda_2\mathbf{\Lambda}'_* + \frac{\lambda_1\sqrt{\lambda_2}}{2}\mathbf{\Lambda}'_*\mathbf{Z}_*\right)^{-1} \\ &= \begin{bmatrix}\mathbf{X}_u^{\mathrm{T}}\mathbf{\Omega}\mathbf{X}_u & \mathbf{X}_u^{\mathrm{T}}\mathbf{\Omega}\mathbf{X}_r \\ \mathbf{X}_r^{\mathrm{T}}\mathbf{\Omega}\mathbf{X}_u & \mathbf{X}_r^{\mathrm{T}}\mathbf{\Omega}\mathbf{X}_r + \lambda_2\mathbf{\Lambda}' + \frac{\lambda_1\sqrt{\lambda_2}}{2}\mathbf{\Lambda}'\mathbf{Z}\end{bmatrix}^{-1}. \end{aligned} \tag{18}$$

With the choice of blocks as in (18), blockwise inversion renders the largest required matrix inverse $(\mathbf{X}_r^{\mathrm{T}}\mathbf{\Omega}\mathbf{X}_r + \lambda_2\mathbf{\Lambda}' + \frac{\lambda_1\sqrt{\lambda_2}}{2}\mathbf{\Lambda}'\mathbf{Z})^{-1}$, of dimension $p \times p$. Inversion of this matrix is done efficiently by applying the Woodbury identity.

9.2 Monotonicity of the penalty parameters

Enforcing monotonicity of the penalty multipliers is desirable in some settings. An example of such a setting is if we have p-values from a previous, related study available. A p-values-based partitioning of the variables may, *a priori*, be expected to render penalty multipliers that increase monotonically with p-value. That is, larger p-values may be expected to yield at least as large penalty multipliers as variables with smaller p-values. We propose to include this *a priori* assumption by requiring the penalty multipliers to increase monotonically with p-value, thereby also stabilizing their estimates.

A natural way of enforcing monotonicity is to extend the constraint in (10) in the Main Document with $\lambda'_1 \leq \dots \leq \lambda'_G$. The problem is still convex and may be numerically solved. From experience, however, we note that in combination with this constrained optimisation, the EM algorithm described in Section 4 in the MD often converges to a local optimum close to the initialisation. We

therefore enforce monotonicity through a *post hoc* isotonic regression on the penalty multipliers after every optimisation step.

9.3 Multiple partitions

In many cases the features may be partitioned in more than one way. For example, we may have both information on annotation and p-values from a previous study available. A naive way of incorporating multiple partitions is to cross-tabulate the partitions and create a separate group for every combination. This poses two problems: (i) The number of penalty parameters increases exponentially with the number of partitions and (ii) some of these combinations may contain only few features, so that the estimation procedure becomes unstable. We propose to stabilise the procedure and keep the number of parameters to estimate manageable by modelling the penalty parameters multiplicatively.

We describe our implementation here for two partitions of the features. To this end, let $(\mathcal{G}_1(1), \dots, \mathcal{G}_1(G_1))$ and $(\mathcal{G}_2(1), \dots, \mathcal{G}_2(G_2))$ denote the two partitions, containing G_1 and G_2 groups respectively. Furthermore, in the following we assume that the empty sum and empty product evaluate to 0 and 1, respectively. In this two-partition setting we have two penalty multipliers per feature, represented by λ'_{g_1} and λ''_{g_2}, respectively. The generalised frequentist elastic net estimator and corresponding conditional prior are now:

$$\hat{\boldsymbol{\beta}} := \underset{\boldsymbol{\beta}}{\operatorname{argmax}}\, \ell(\mathbf{y};\boldsymbol{\beta}) - \frac{\lambda_1}{2}\sum_{g_1=1}^{G_1}\sum_{g_2=1}^{G_2}\sqrt{\lambda'_{g_1}\lambda''_{g_2}}\sum_{\substack{j\in\mathcal{G}_1(g_1)\\ \cap\mathcal{G}_2(g_2)}}|\beta_j| - \frac{\lambda_2}{2}\sum_{g_1=1}^{G_1}\sum_{g_2=1}^{G_2}\lambda'_{g_1}\lambda''_{g_2}\sum_{\substack{j\in\mathcal{G}_1(g_1)\\ \cap\mathcal{G}_2(g_2)}}\beta_j^2, \tag{19a}$$

$$\boldsymbol{\beta}|\boldsymbol{\tau} \sim \prod_{g_1=1}^{G_1}\prod_{g_2=1}^{G_2}\prod_{\substack{j\in\mathcal{G}_1(g_1)\\ \cap\mathcal{G}_2(g_2)}}\mathcal{N}\left(0, \frac{1}{\lambda'_{g_1}\lambda''_{g_2}\lambda_2}\frac{\tau_j-1}{\tau_j}\right). \tag{19b}$$

After switching to the parametrisation in Friedman et al. (2010), the new penalty multiplier estimates $\boldsymbol{\lambda}' = \begin{bmatrix}\lambda'_1 & \cdots & \lambda'_{G_1}\end{bmatrix}^{\mathrm{T}}, \boldsymbol{\lambda}'' = \begin{bmatrix}\lambda''_1 & \cdots & \lambda''_{G_2}\end{bmatrix}^{\mathrm{T}}$ are given by:

$$\begin{aligned}\boldsymbol{\lambda}'^{(k+1)}, \boldsymbol{\lambda}''^{(k+1)} := \underset{\boldsymbol{\lambda}',\boldsymbol{\lambda}''}{\operatorname{argmax}} \Bigg\{ & \frac{1}{2}\sum_{g_1=1}^{G_1}|\mathcal{G}_1(g_1)|\log(\lambda'_{g_1}) + \frac{1}{2}\sum_{g_2=1}^{G_2}|\mathcal{G}_2(g_2)|\log(\lambda''_{g_2}) \qquad (20a)\\ & - \frac{(1-\alpha)\lambda}{4}\sum_{g_1=1}^{G_1}\sum_{g_2=1}^{G_2}\lambda'_{g_1}\lambda''_{g_2}d^{(k)}_{g_1g_2}\Bigg\}\\ \text{subject to } & \prod_{g_1=1}^{G_1}\prod_{g_2=1}^{G_2}(\lambda'_{g_1}\lambda''_{g_2})^{|\mathcal{G}_1(g_1)\cap\mathcal{G}_2(g_2)|} = 1, \qquad (20b)\end{aligned}$$

with the $d_{g_1g_2}$ terms calculated as $\sum_{\substack{j\in\mathcal{G}_1(g_1)\\ \cap\mathcal{G}_2(g_2)}}\left[\boldsymbol{\Sigma}^{(k)}_{jj} + (\boldsymbol{\mu}^{(k)}_j)^2\right]\left(1+\alpha\lambda^{1.5}\sqrt{\frac{1-\alpha}{8\chi^{(k)}_j}}\right)$. The optimisation in (20) is again a convex problem that is easily solved by some numerical optimisation routine. This method naturally generalises to more than two partitions of the data.

In (19) we model the two partition-specific penalty multipliers in a multiplicative way, mainly for convenience. First, there are computational reasons: The separation of the square root in the L_1-norm penalty term into two terms facilitates the numerical estimation. Additionally, multiplicative modelling of the partitions allows for more flexibility in the estimates. To see this consider the

obvious alternative: additive partition-specific multipliers. Since the penalty multipliers are strictly positive, the constraint on the geometric mean makes one large additive penalty estimate difficult to compensate with a smaller estimate, thereby impairing flexibility of the model. Large multiplicative penalty multipliers are easier to compensate for by smaller penalty multipliers, rendering this the less restrictive option for modelling the partitions.

10 Empirical-variational Bayes for the ridge and lasso

10.1 Ridge regression

Ridge regression is a special case of the elastic net, that does not include an L_1-norm on the parameters. Its estimator is given by:

$$\hat{\boldsymbol{\beta}} := \underset{\boldsymbol{\beta}}{\operatorname{argmax}}\, \ell(\mathbf{y};\boldsymbol{\beta}) - \frac{\lambda_2}{2}\left\|\boldsymbol{\beta}\right\|_2.$$

The Bayesian version of ridge regression is regression under a Gaussian prior. The generalised version of this prior is:

$$\boldsymbol{\beta} \sim \prod_{g=1}^{G}\prod_{j\in\mathcal{G}(g)} \mathrm{N}(0,(\lambda_2\lambda'_g)^{-1}).$$

Although the posterior is analytically calculated in the linear case, in the logistic case we must approximate it iteratively. The variational distributions in this case are:

$$\begin{aligned} q^*_{\boldsymbol{\beta}}(\boldsymbol{\beta}) &\sim \mathcal{N}(\boldsymbol{\mu},\boldsymbol{\Sigma}),\\ q^*_{\boldsymbol{\omega}}(\boldsymbol{\omega}) &\sim \prod_{i=1}^{n}\mathcal{PG}(m_i,c_i). \end{aligned}$$

The parameters c_i and $\boldsymbol{\mu}$ remain unchanged compared to the elastic net and are updated, together with $\boldsymbol{\Sigma}$, until convergence:

$$\boldsymbol{\Sigma}^{t+1} = (\mathbf{X}^{\mathrm{T}}\boldsymbol{\Omega}^{(t)}\mathbf{X}+\lambda_2\boldsymbol{\Lambda}')^{-1}, \text{ with } \boldsymbol{\Omega}^{(t)} = \operatorname{diag}\left[\frac{m_i}{2c_i^{(t)}}\tanh\left(\frac{c_i^{(t)}}{2}\right)\right].$$

Updating the penalty parameters is done by solving the following (convex) constraint optimisation problem:

$$\begin{aligned} \boldsymbol{\lambda}'^{(k+1)} &= \underset{\boldsymbol{\lambda}'}{\operatorname{argmax}}\,\frac{1}{2}\sum_{g=1}^{G}|\mathcal{G}(g)|\log(\lambda'_g) - \frac{\lambda_2}{2}\sum_{g=1}^{G}\lambda'_g d_g^{(k)}\\ &\text{subject to } \prod_{g=1}^{G}(\lambda'_g)^{|\mathcal{G}(g)|} = 1, \end{aligned}$$

with the terms $d_g^{(k)}$ given by:

$$d_g^{(k)} = \sum_{j\in\mathcal{G}(g)}((\boldsymbol{\mu}_j^{(k)})^2+\boldsymbol{\Sigma}_{jj}^{(k)}).$$

10.2 Lasso regression

In lasso regression, another special case of the elastic net, we have the following estimator:

$$\hat{\boldsymbol{\beta}} := \underset{\boldsymbol{\beta}}{\text{argmax}}\, \ell(\mathbf{y};\boldsymbol{\beta}) - \frac{\lambda_1}{2} \|\boldsymbol{\beta}\|_1 .$$

The Bayesian counterpart is regression under a Laplace prior on the model parameters. The generalised Bayesian prior may decomposed as:

$$\begin{aligned}
\boldsymbol{\beta}|\boldsymbol{\tau} &\sim \prod_{j=1}^{p} \mathcal{N}(0, \tau_j),\\
\boldsymbol{\tau} &\sim \prod_{g=1}^{G} \prod_{j \in \mathcal{G}(g)} \text{Exp}\left(\frac{\sqrt{\lambda'_g}\lambda_1^2}{8}\right).
\end{aligned}$$

The variational distributions are similar as in the elastic net, except that we do not re-parametrise the τ_j:

$$\begin{aligned}
q_{\boldsymbol{\beta}}^*(\boldsymbol{\beta}) &\sim \mathcal{N}(\boldsymbol{\mu}, \boldsymbol{\Sigma}),\\
q_{\boldsymbol{\omega}}^*(\boldsymbol{\omega}) &\sim \prod_{i=1}^{n} \mathcal{PG}(m_i, c_i),\\
q_{\boldsymbol{\tau}}^*(\boldsymbol{\tau}) &\sim \prod_{g=1}^{G} \prod_{j \in \mathcal{G}(g)} \mathcal{GIG}\left(\frac{1}{2}, \phi_g, \chi_j\right).
\end{aligned}$$

The parameters c_i and $\boldsymbol{\mu}$ and remain unchanged compared to the elastic net, while the ϕ_g are given by:

$$\phi_g = \lambda_g \frac{\lambda_1^2}{4}.$$

The following parameters are updated, together with the unchanged c_i and $\boldsymbol{\mu}$, until convergence:

$$\begin{aligned}
\boldsymbol{\Sigma}^{(t+1)} &= (\mathbf{X}^{\text{T}}\boldsymbol{\Omega}^{(t)}\mathbf{X} + \mathbf{Z}^{(t)})^{-1},\\
&\quad \text{with } \boldsymbol{\Omega}^{(t)} = \text{diag}\left[\left(\frac{m_i}{2c_i^{(t)}}\right)\tanh\left(\frac{c_i^{(t)}}{2}\right)\right] \text{ and } \mathbf{Z}^{(t)} = \text{diag}\left(\sqrt{\frac{\phi_g(j)}{\chi_j^{(t)}}}\right),\\
\chi_j^{(t+1)} &= \boldsymbol{\Sigma}_{jj}^{(t+1)} + (\boldsymbol{\mu}_j^{(t+1)})^2, \text{ for } j = 1, \dots, p.
\end{aligned}$$

Updating the penalty parameters is done by solving the following (convex) constraint problem:

$$\begin{aligned}
\boldsymbol{\lambda}'^{(k+1)} = \underset{\boldsymbol{\lambda}'}{\text{argmax}} &\sum_{g=1}^{G} |\mathcal{G}(g)| \log(\lambda'_g) - \frac{\lambda_1^2}{8} \sum_{g=1}^{G} \lambda'_g d_g^{(k)}\\
\text{subject to } &\prod_{g=1}^{G} (\lambda'_g)^{|\mathcal{G}(g)|} = 1,
\end{aligned}$$

with the terms $d_g^{(k)}$ given by:

$$d_g^{(k)} = \sum_{j \in \mathcal{G}(g)} \left(\frac{1}{\phi_g^{(k)}} + \frac{\chi_j^{(k)}}{\phi_g^{(k)}}\right).$$

Note that the ϕ_g are updated after every penalty multiplier update iteration as well, in contrast to the elastic net.

11 Application to MicroRNAs in colorectal cancer

11.1 Additional comparisons

We compared `gren` to two methods in addition to the ones in the MD Section 5.2: the sparse group lasso (`SGL`) by Simon et al. (2013) and the group exponential lasso (`gel`) by Breheny (2015). For all methods with a tuning parameter α, we set $\alpha \in \{0.05, 0.5, 0.95\}$ and present the AUC and Brier skill score on the test set in Figures 1– 2.

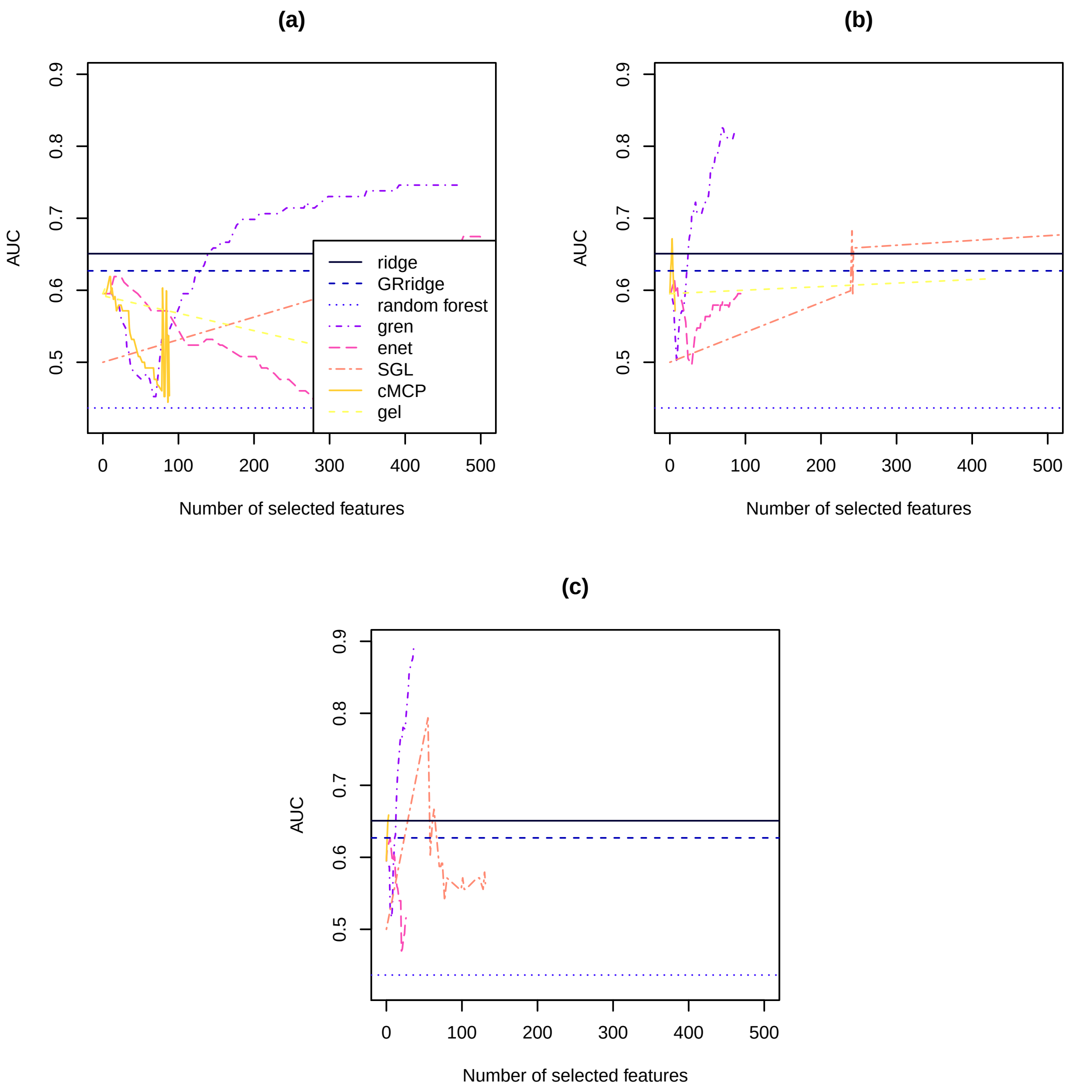

Figure 1: Estimated AUC for the colorectal cancer example with (a) $\alpha = 0.05$, (b) $\alpha = 0.5$, and (c) $\alpha = 0.95$.

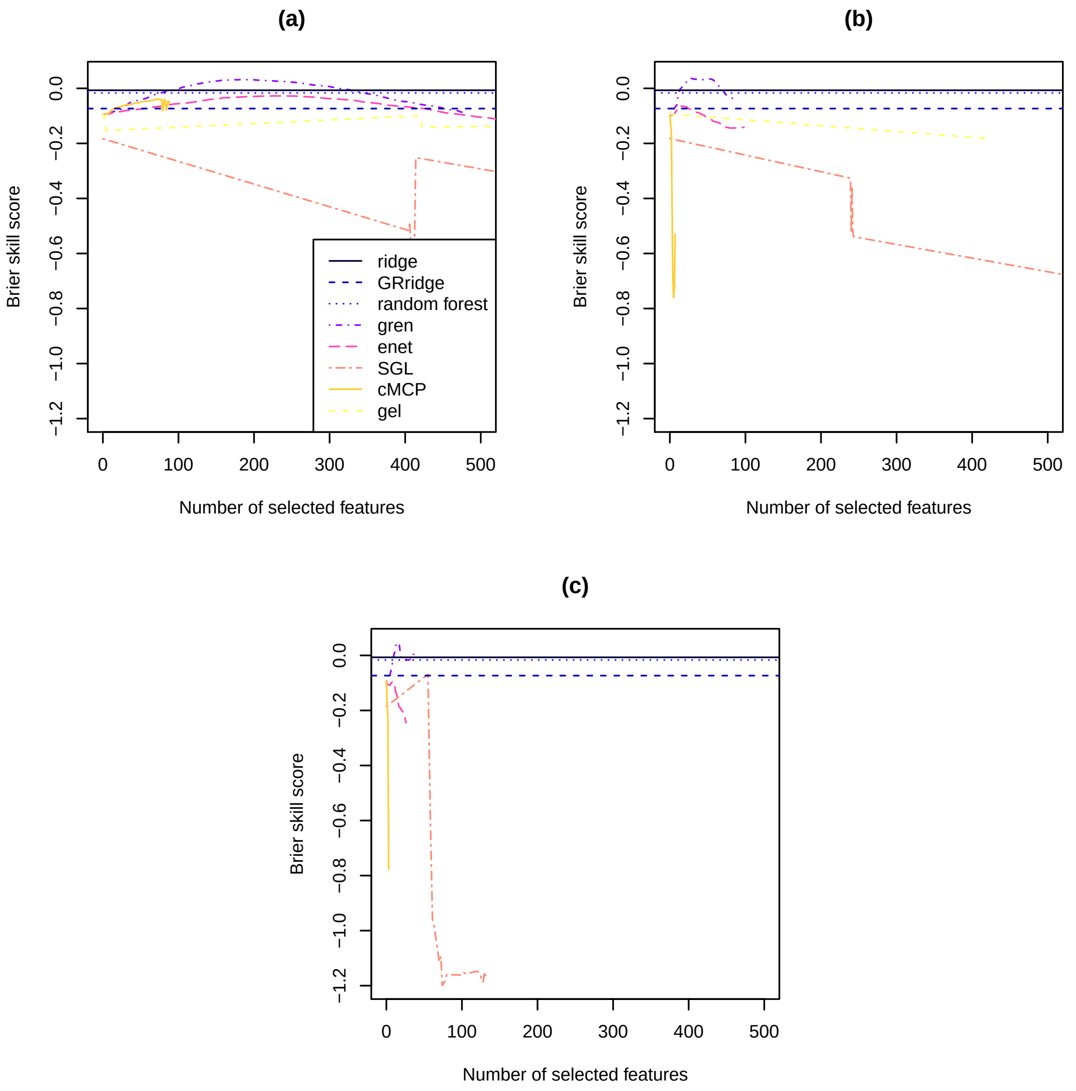

Figure 2: Estimated Brier skill score for the colorectal cancer example with (a) $\alpha = 0.05$, (b) $\alpha = 0.5$, and (c) $\alpha = 0.95$.

11.2 Random groups

Considering that the features may be partitioned into many groups, with one parameter per group, we have to be aware of overfitting risks. We investigated this using the data introduced in Section 5.2 of the MD, randomly dividing the features into three groups. We fixed the group sizes to the group sizes used in the MD. Under this random partitioning of the features, we expect all penalty multipliers to be estimated as one if no overfitting occurs.

We compared the estimated multipliers to the estimates by `GRridge`. Since these results depend on one specific randomisation of the groups, we repeated the procedure 100 times and present the

results in Figure 3. From this figure we see that the estimates for `gren` are relatively close to one. The estimates by `GRridge` show more variation. We found no strong indication of bias, with median estimated penalty parameters 1, 1, 0.99 for `GRridge` and 1, 1, 0.99; 1, 1.01, 0.99, and 1, 1.01, 0.98 for `gren` with $\alpha \in \{0.05, 0.5, 0.95\}$, respectively.

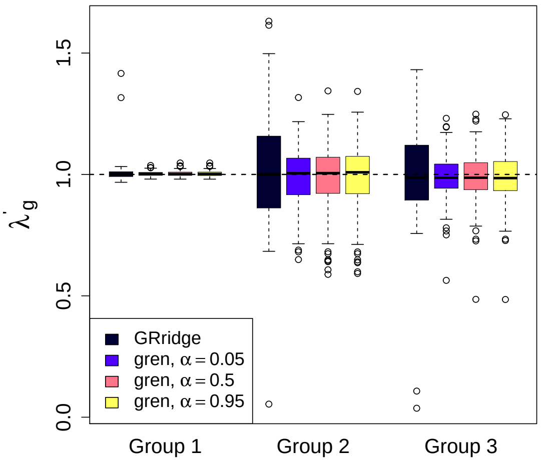

Figure 3: Results for the colorectal cancer example with 100 realisations of 3 random groups of features.

We repeated the simulation with ten evenly sized groups. Figure 4 presents the estimated penalty multipliers for 100 random splits of the microRNA features into 10 evenly sized groups for `GRridge` and `gren` with $\alpha \in \{0.05, 0.5, 0.95\}$. From the Figure we see that `GRridge` shows more variation around one then the other models. There is no indication of bias: the median penalty multiplier for `GRridge` is 1, while the median multipliers in `gren` are 1, 1, 1.01 for $\alpha \in \{0.05, 0.5, 0.95\}$, respectively.

11.3 Stability of selection

One common criticism on the elastic net, and especially the lasso, is the instability of feature selection. That is, different data sets yield different sets of selected features. To investigate the stability of feature selection by `gren` we created 50 stratified bootstrap samples from the microRNA data (Neerincx et al., 2018), where we stratified by the target of prediction, treatment response. For each of these bootstrap samples we estimated the regular elastic net and `gren` for $\alpha \in \{0.05, 0.5, 0.95\}$, where we selected 25 features (including the 5 unpenalized covariates). We calculated the size of all $\binom{50}{2}$ intersections of selected features and present the results in Figure 5.

12 Application to RNAseq in oral cancer

In addition to the results presented in Section 5.3 of the MD, we compared `gren` to the sparse group lasso (`SGL`) by Simon et al. (2013) and the group exponential lasso (`gel`) by Breheny (2015). The

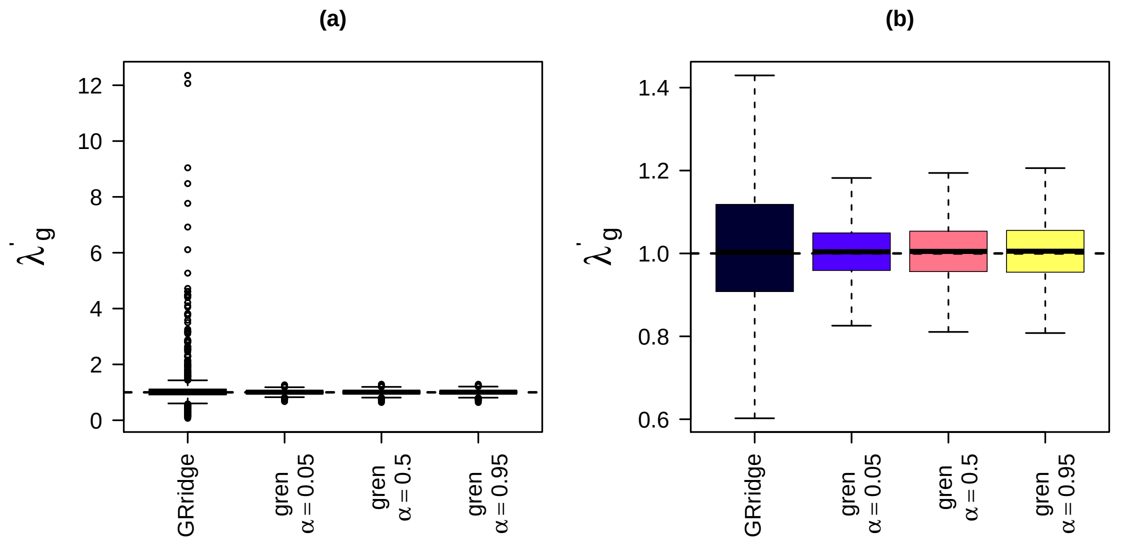

Figure 4: Results for the colorectal cancer example with 100 realisations of 10 random groups of features, plotted (a) with outliers, and (b) without outliers.

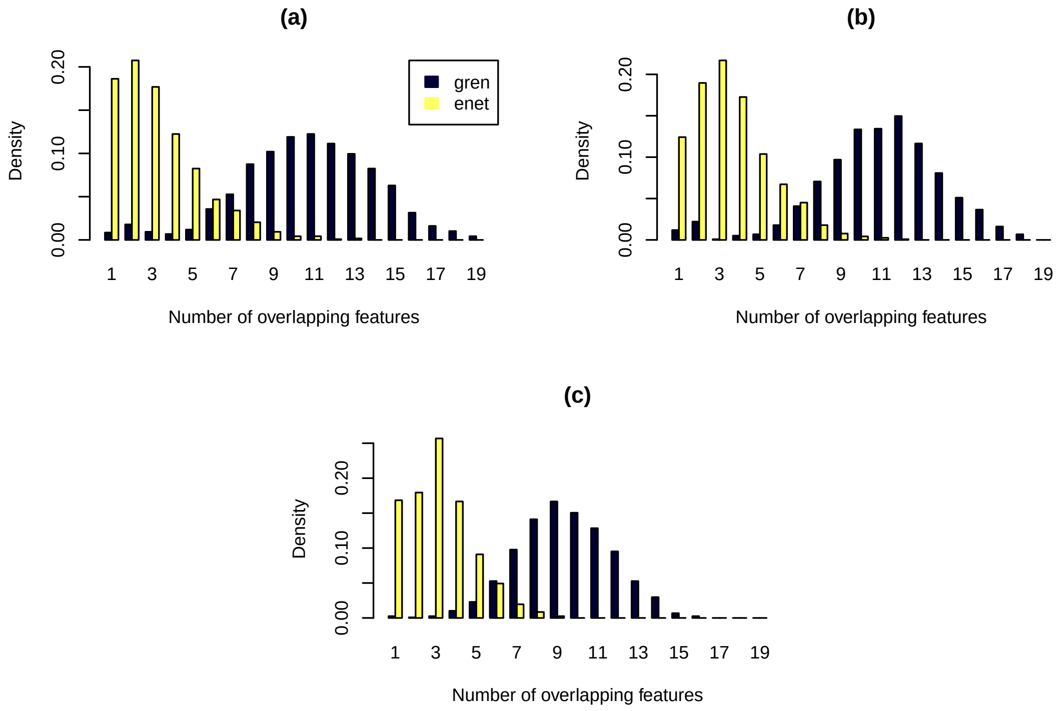

Figure 5: Stability of feature selection in colorectal cancer example for (a) $\alpha = 0.05$, (b) $\alpha = 0.5$, and (c) $\alpha = 0.95$.

original group lasso methods are not designed to deal with overlapping groups, so in addition to using the versions that allow for overlapping groups, we cross-tabulated the two co-data partitions to create one paritioning with 25 groups for `cMCP` and `gel`. We use $\alpha \in \{0.05, 0.5, 0.95\}$ for the methods that require this extra tuning parameter. The AUCs and Brier skill scores calculated on the validation set are presented in Figures 6– 7

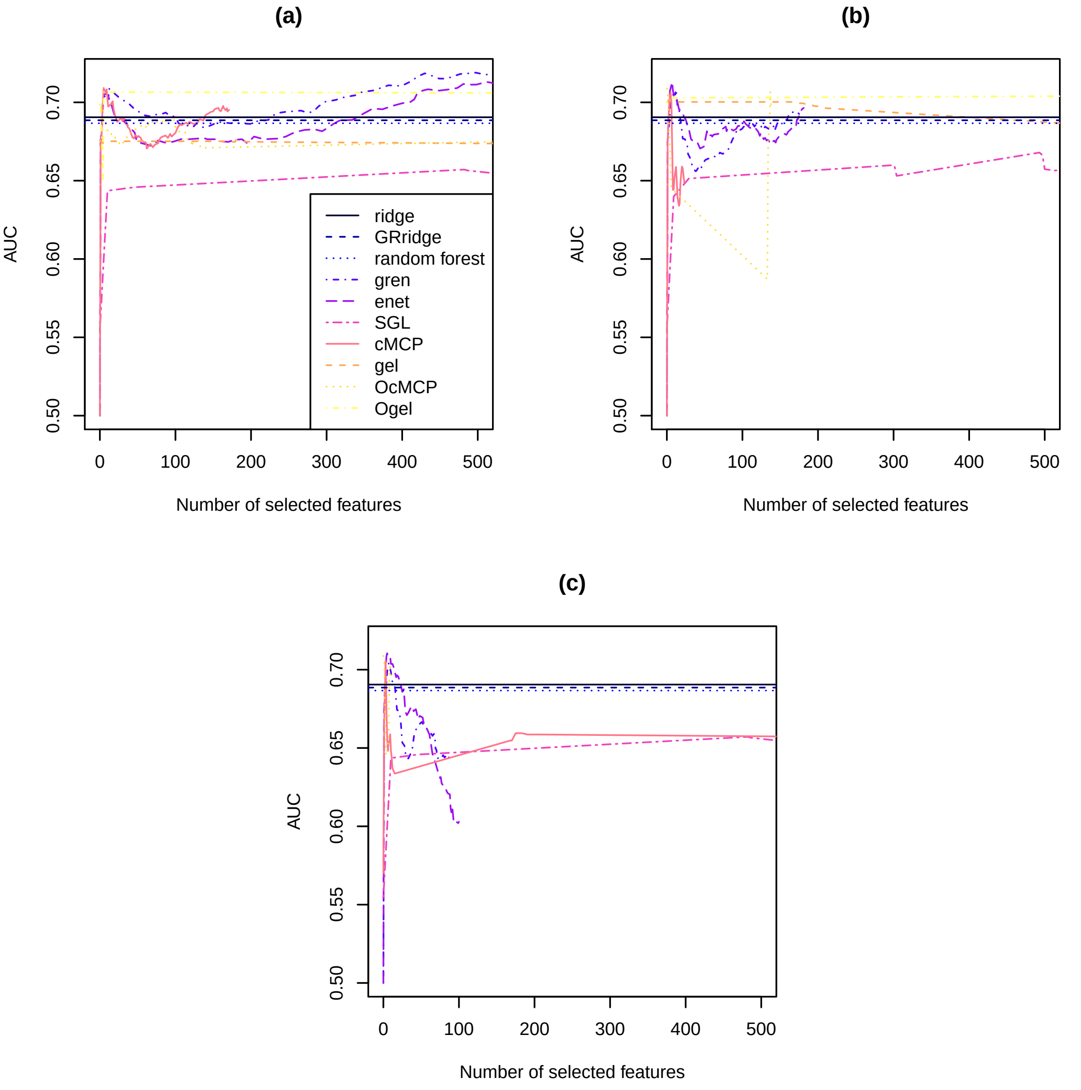

Figure 6: Estimated AUC for the oral cancer example with (a) $\alpha = 0.05$, (b) $\alpha = 0.5$, and (c) $\alpha = 0.95$.

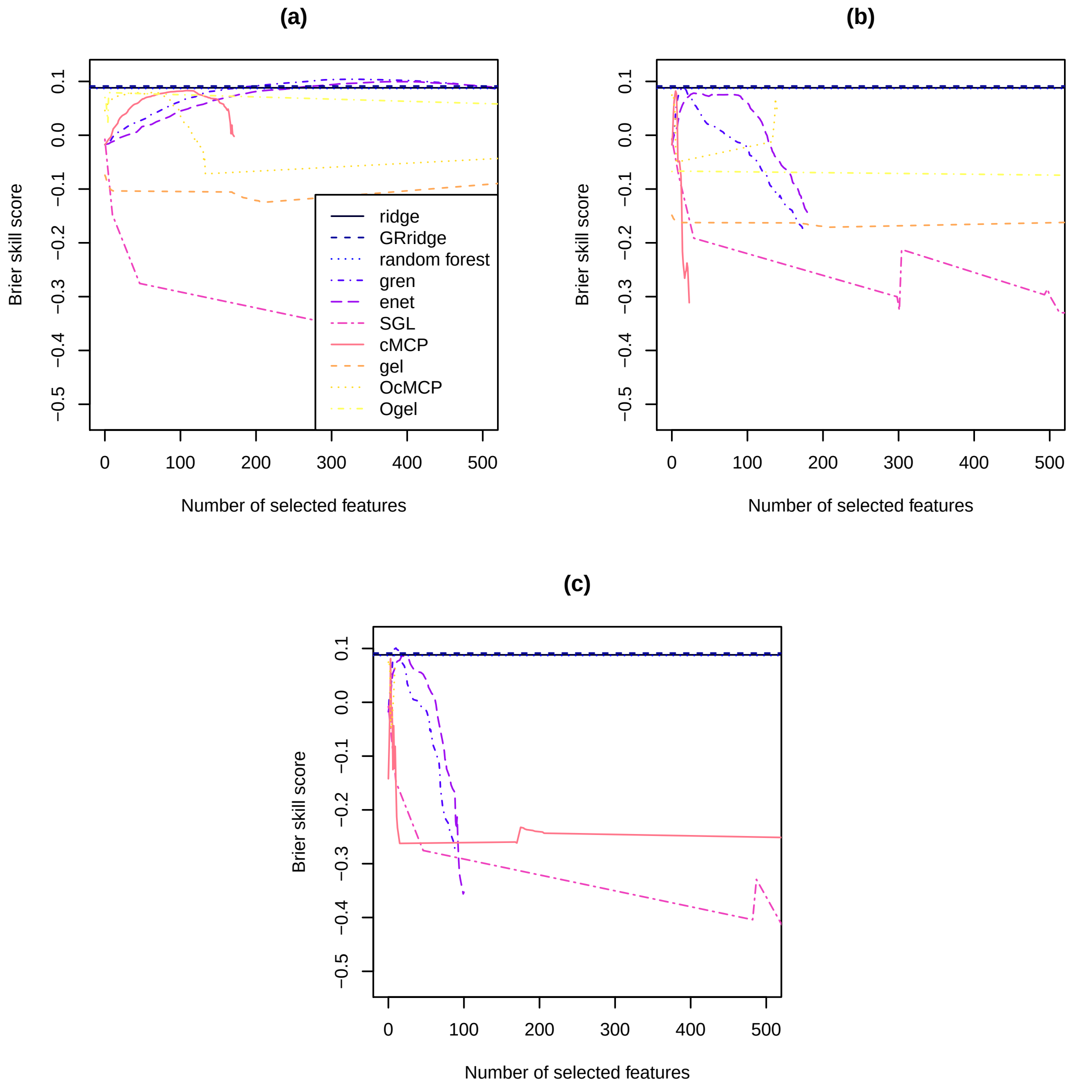

Figure 7: Estimated Briers skill score for the oral cancer example with (a) $\alpha = 0.05$, (b) $\alpha = 0.5$, and (c) $\alpha = 0.95$.

13 Application to metabolomics in alzheimer

We applied our method to an Alzheimer's dataset from de Leeuw et al. (2017). The data consist of 87 diagnosed Alzheimer's patients with the $\epsilon 4$ allele of the APOE gene and 87 subjects with subjective un-diagnosed cognitive decline, without the $\epsilon 4$ allele. The aim of the study was to diagnose subjects with Alzheimer's disease based on their metabolic profile. After pre-processing, 230 metabolite expression levels were obtained. To enhance classification performance, we included two sources of co-data: (a) the relative standard devitation of the measurements (RSD), a commonly used quality score (smaller is better) for metabolites, binned into five groups, and (b) the

node degree of the metabolites, based on a differential network analysis of the metabolites, binned into three categories: 0 degree, positive, lower than average degree, and postive, higher than average degree. We expect a lower RSD and a higher node degree to be indicative of an important metabolite and therefore receive less penalty.

To estimate performance measures AUC and Briers skill score, we split the data into 120 training instances and 54 test instances. The training data is used to fit the models, while the test data is used to estimate the performance measures on. This resulted in the estimated multipliers and performance measures presented in Figures 8– 10.

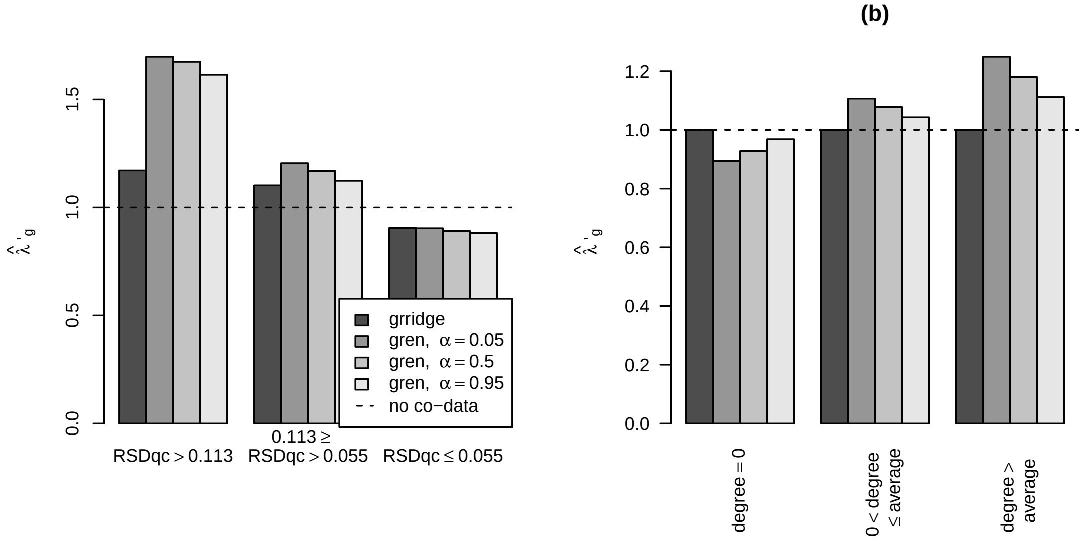

Figure 8: Estimated penalty multiplies for the (a) quality score and the (b) network degree in the alzheimer example.

In this example, `GRridge` and `gren` underperform due to: (i) a small number of features to learn the penalty parameters from and (ii) Large negative correlations between the metabolites (see Figure 11). Both (i) and (ii) make estimation of the penalty multipliers difficult. The other methods estimate one global penalty parameter, so do not suffer from the lower dimensionality of the problem and the large negative correlation.

14 Application to microRNAs in cervical cancer

A deep sequencing analysis on small non-coding ribonucleic acid (microRNAseq) was performed on 24 women with high-grade cervical intraepithelial neoplasia (CIN3) and 32 healthy women for the purpose of finding relevant screening markers for cervical cancer screening. The next generation sequencing analysis resulted in 2576 transcripts. The data was normalized and pre-processed, rendering 752 transcripts. More details of the data sets and the pre-processing steps are found in the supplementary material of Novianti et al. (2017).

The 752 microRNAs were divided in three classes based on their conservation status. Conservation status refers to whether the microRNAs are only found in humans or are conserved across different species. They come in three classes: (a) non-conserved (535), (b) conserved in mammals (70),

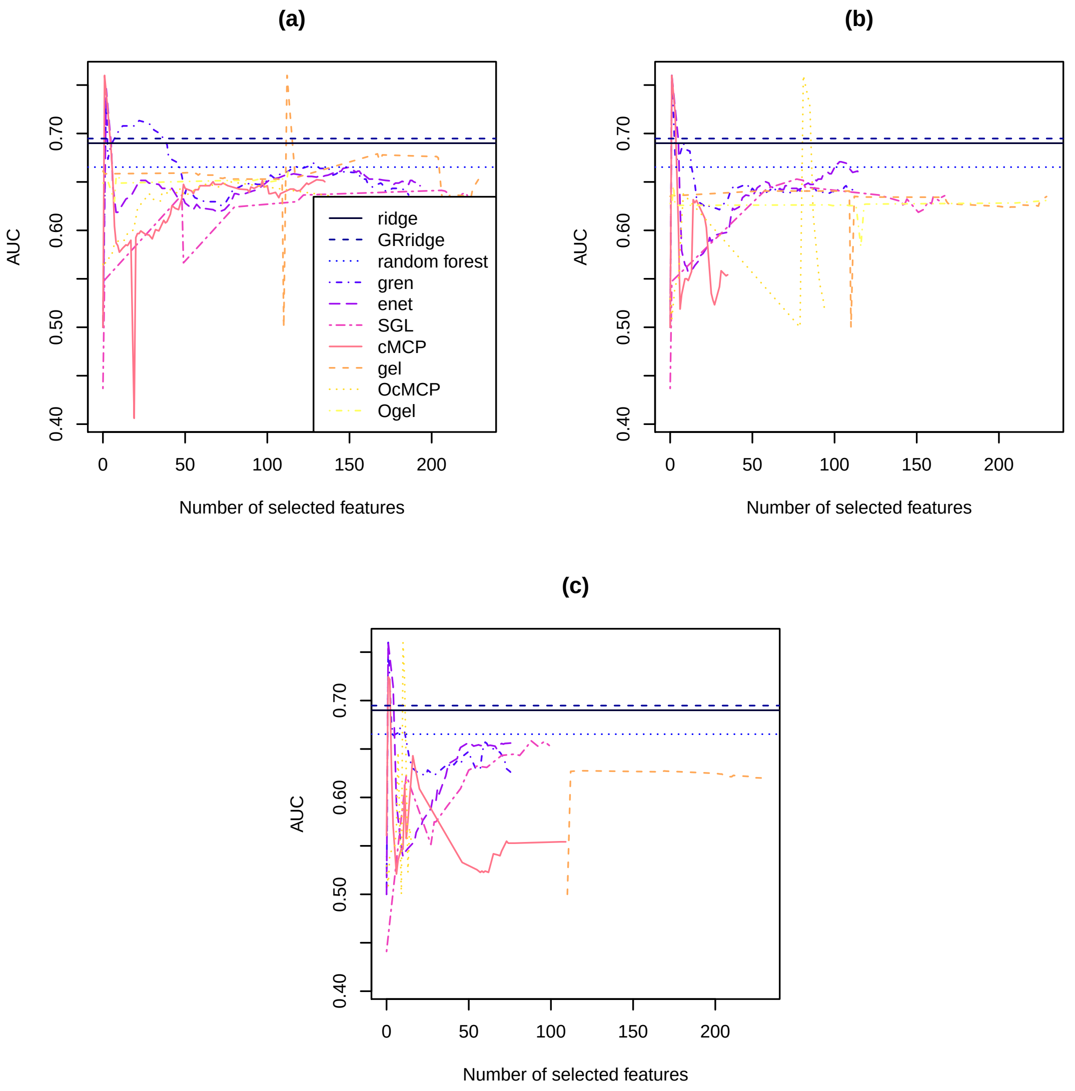

Figure 9: Estimated AUC for the alzheimer example with with (a) $\alpha = 0.05$, (b) $\alpha = 0.5$, and (c) $\alpha = 0.95$.

and (c) conserved across most vertebrates (147). We expect (c) to contain the most important microRNAs, followed by (b) and (a), because these microRNAs have not been lost during evolution and might therefore regulate important processes.

The estimated multipliers, AUC, and Brier skill score are in Figures 12– 14, respectively. In this example the group regularized methods outperform the regular methods with respect to discrimination (AUC) and calibration (Brier skill score).

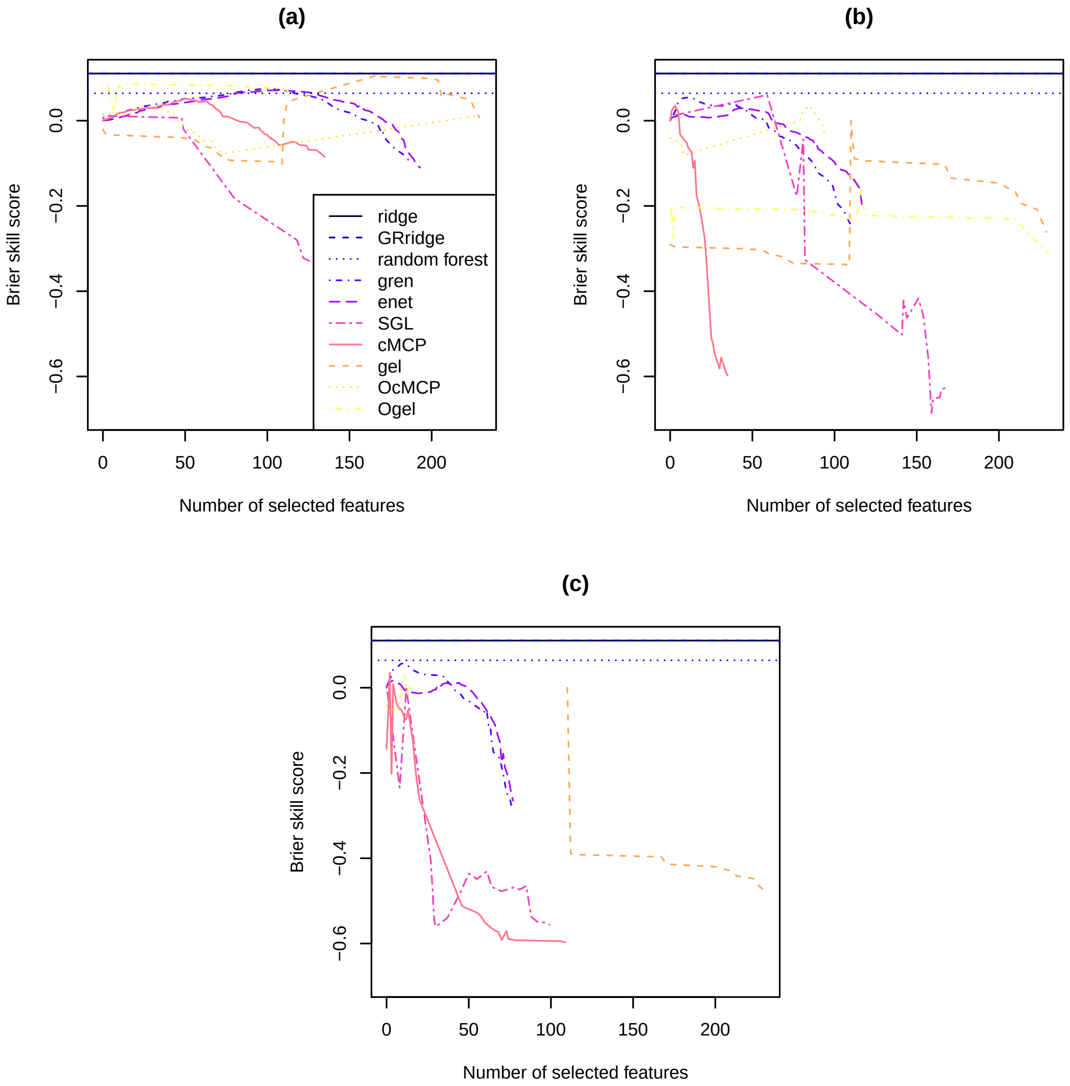

Figure 10: Estimated Brier skill score for the alzheimer example with (a) $\alpha = 0.05$, (b) $\alpha = 0.5$, and (c) $\alpha = 0.95$.

15 Simulations

We simulated data according to five different scenarios that are explained in more detail below. For the regular elastic net, `gren`, `cMCP`, and `gel`, we fix $\alpha \in \{0.05, 0.5, 0.95\}$. In `gren` and the elastic net, α determines the proportion of L_1-norm penalty, such that the first setting closely resembles the ridge setting, where $\alpha = 0$, while the third setting is similar to the lasso with $\alpha = 1$. In `cMCP` and `gel`, α determines the ratio of group-level penalization ($\alpha = 0$) to feature level ($\alpha = 1$) penalization. For the methods that do automatic feature selection, we estimate models for a range of model sizes: $\hat{p} \in \{2, 4, 8, 16, 32, 64, 128, 256\}$.

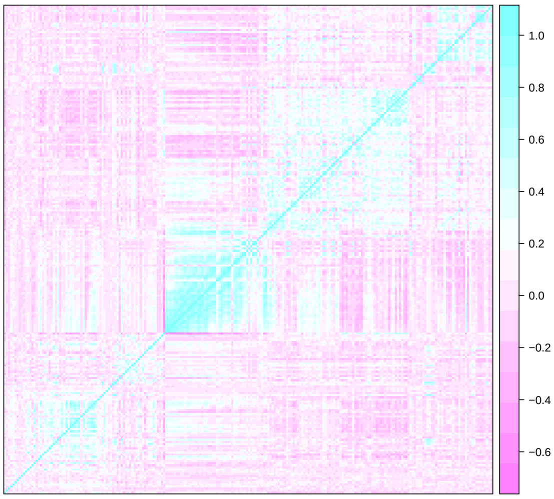

Figure 11: Correlations between the metabolites.

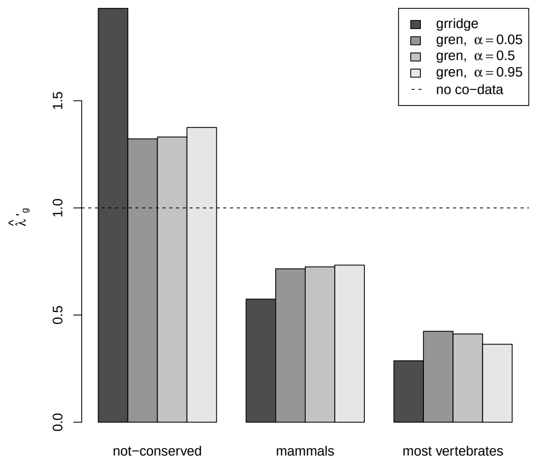

Figure 12: Estimated penalty multiplies for conservation status in cervical cancer example.

In all scenarios we created a training set of $n = 100$ samples to estimate the models and a test set of $n_{\text{test}} = 1000$ samples to compute the performance measures. The features $\mathbf{x}_i$, $i = 1, \dots, n$, are taken from a p-dimensional zero-centred Gaussian distribution, where p varies from $p = 900$ to $p = 1000$. The covariance matrix of the features and simulation of the model parameters β_j, $j = 1, \dots, p$ varies between Scenarios. We simulated the outcomes y_i, $i = 1, \dots, n$, from a binary logistic model: $y_i \sim \mathcal{B}(1, \text{expit}(\mathbf{x}_i^{\text{T}} \boldsymbol{\beta}))$. To mitigate the influence of random variation, we repeated each scenario 100 times and report average area under the receiver operator curve (AUC) and Brier skill score in Figures 15- 19. AUC and Brier skill score are measures of discrimination and

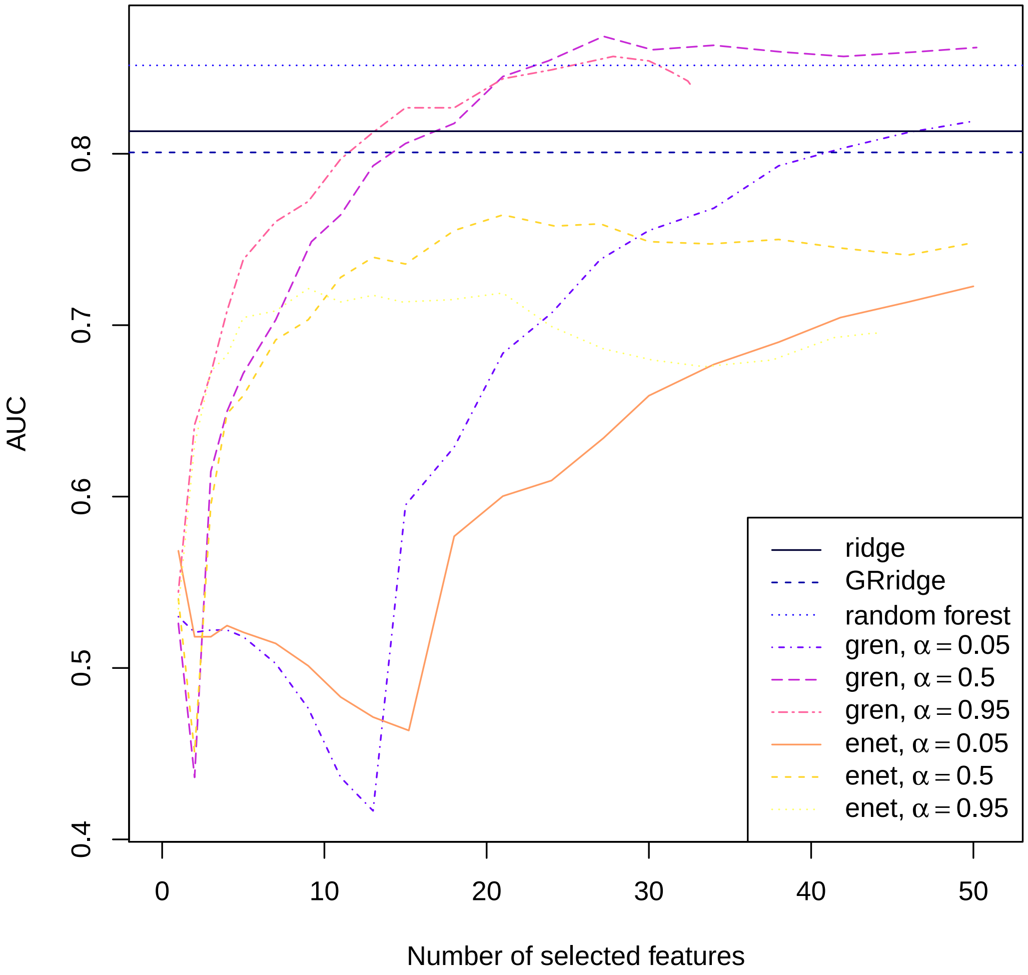

Figure 13: Cross-validated AUC in cervical cancer example

calibration, respectively, and for both a higher value indicates better predictive performance. For the methods with an α parameter, we display the best-performing α. Results for the other α's are presented in Figures 20- 24-

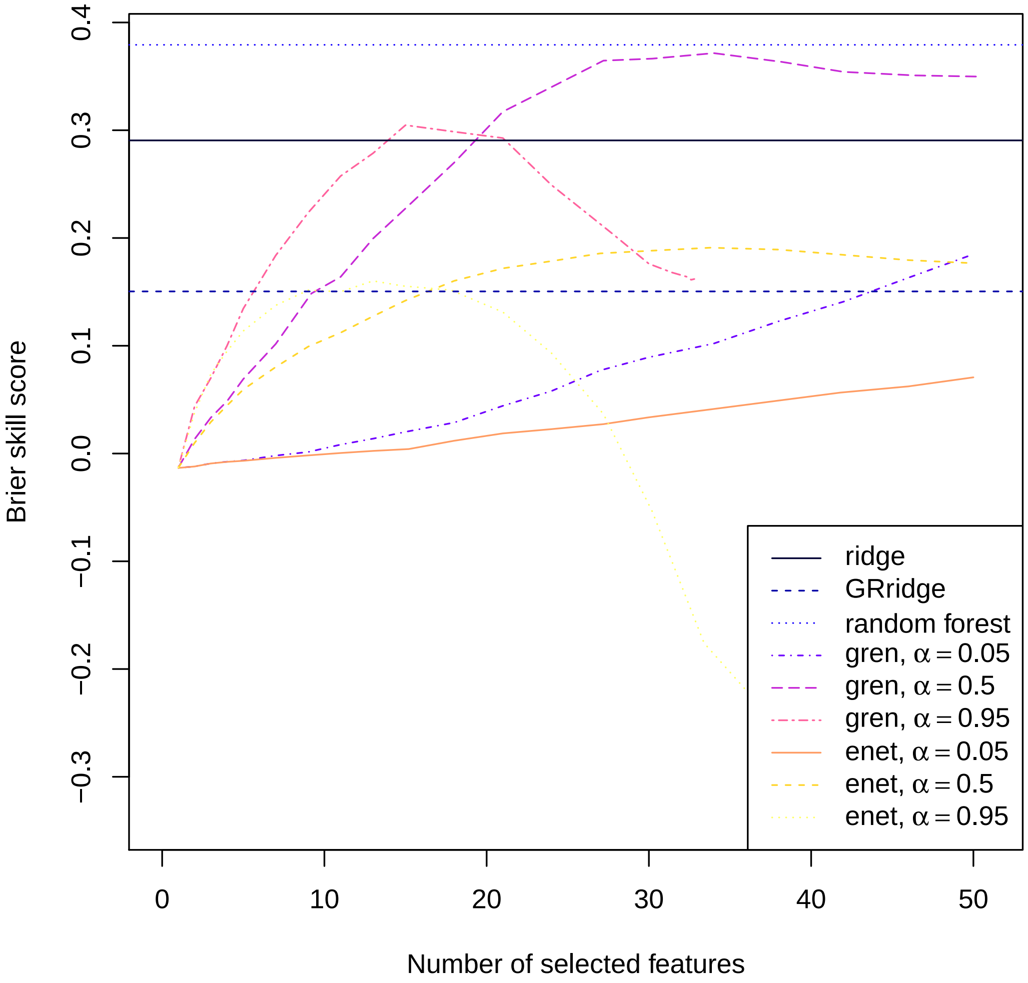

Figure 14: Cross-validated Brier skill score in cervical cancer example

15.1 Scenario 1

In this sparse scenario we assume differential signal between 5 groups of features. The average signal in a group increases by a factor 1.6 with group number:

$$\boldsymbol{\beta} = \big[\underbrace{\mathbf{0} \;\cdots\; \mathbf{0}}_{150} \;\; \underbrace{\beta_1^1 \;\cdots\; \beta_{50}^1}_{50} \;\cdots\; \underbrace{\mathbf{0} \;\cdots\; \mathbf{0}}_{150} \;\; \underbrace{\beta_1^5 \;\cdots\; \beta_{50}^5}_{50}\big]^{\text{T}},$$
$$\beta_j^g \sim \mathcal{U}(0, b_1 \cdot 1.6^{g-1}),\; g = 1, \dots, 5,\; j = 1, \dots, 50,$$

where $\mathcal{U}(a, b)$ denotes the uniform distribution on (a, b). We set $b_1 \approx 0.18$ such that the mean expected signal $\overline{\mathbb{E}(\beta_j^g)} = 0.07$. Furthermore, we introduce correlation between the features by

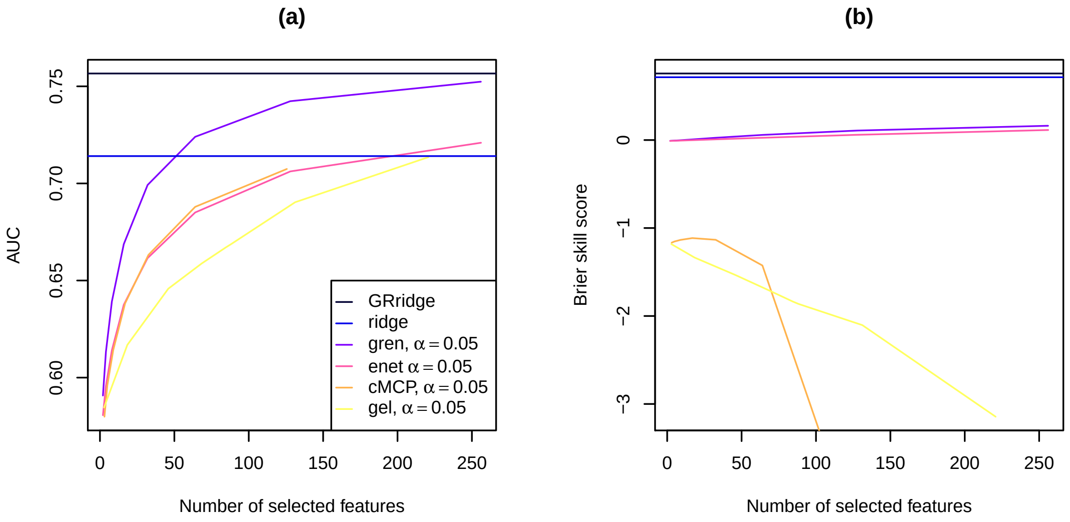

Figure 15: Average (a) AUC and (b) Brier skill score in simulation Scenario (i).

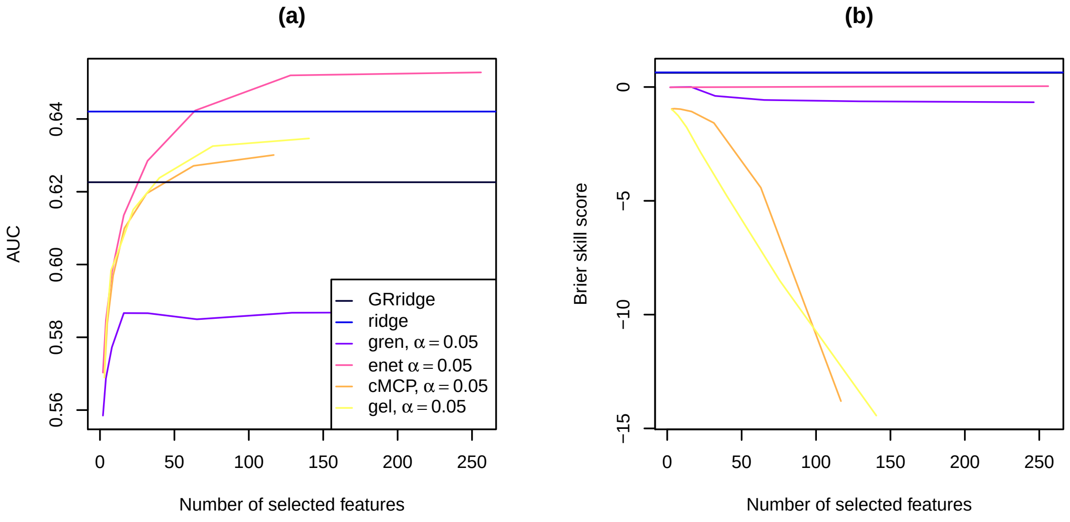

Figure 16: Average (a) AUC and (b) Brier skill score in simulation Scenario (ii).

setting the covariances $\mathbf{\Sigma}_{ij} = 0.5^{|i-j|}$.The model parameters clearly do not follow the relatively heavy-tailed elastic net distribution. Because of this mismatch between the assumed and true distributions, we expect poor performance by `gren`.

15.2 Scenario 2

For this scenario we loosely follow the simulation setup in Meier et al. (2008). We create $G = 300$ groups of features and $p = 900$ features in total, such that we have 3 features per group. There

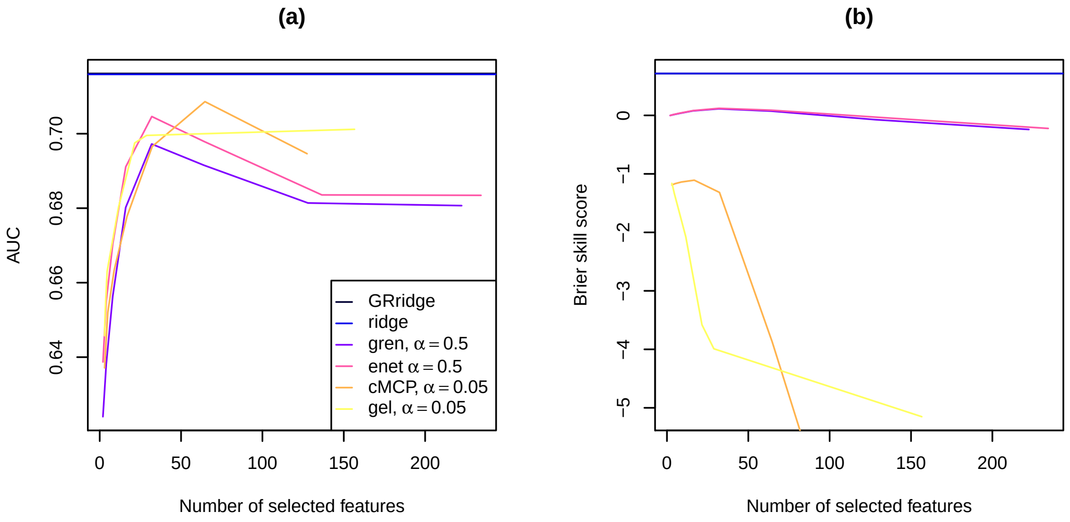

Figure 17: Average (a) AUC and (b) Brier skill score in simulation Scenario (iii).

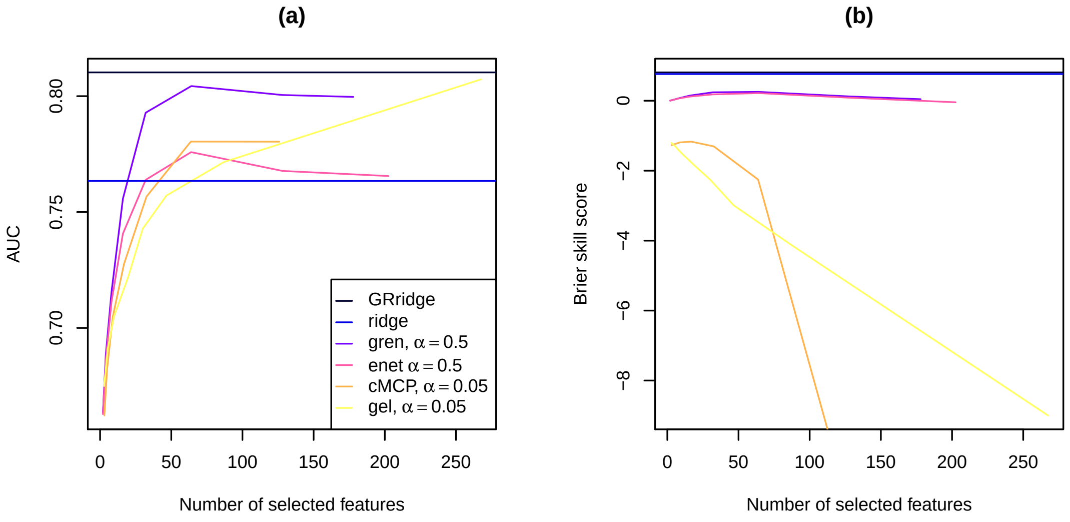

Figure 18: Average (a) AUC and (b) Brier skill score in simulation Scenario (iv).

are 50 active groups, with 2 active parameters with the same value and one inactive parameter per group, such that in total we have 100 non-zero model parameters. The active model parameters are fixed and decrease linearly from 30/51 to 3/510, such that the mean parameter $\overline{\beta}_j = 1/30$. In this example, we included an intercept; $\beta_0 = 10$.

The predictors are sampled in such a way that they represent dummy variables for categorical variables. We start with sampling 300 latent variables: $\mathbf{z} \sim \mathcal{N}(\mathbf{0}, \mathbf{\Sigma})$, with $\mathbf{\Sigma}_{ij} = 0.5^{|i-j|}$, $i, j =$

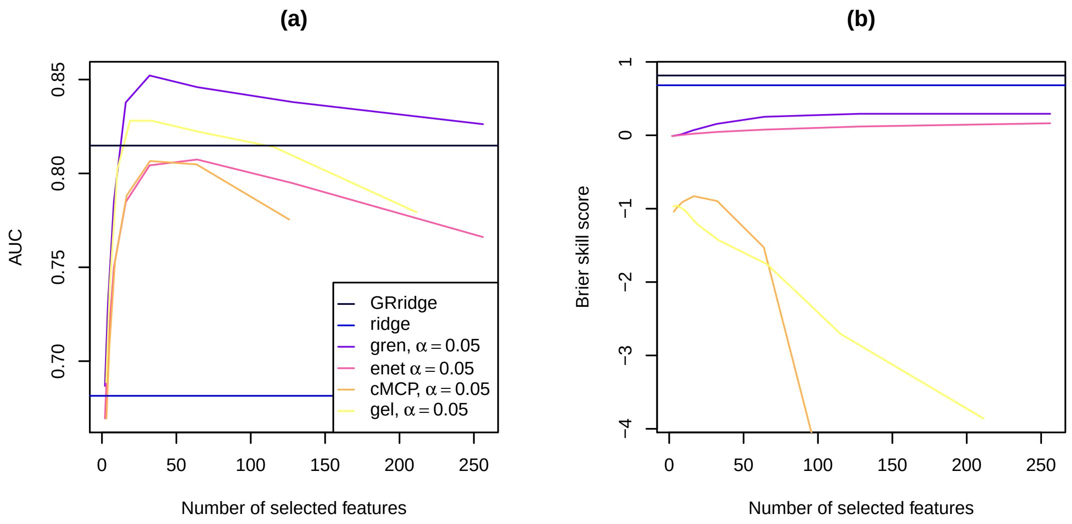

Figure 19: Average (a) AUC and (b) Brier skill score in simulation Scenario (v).

$1, \dots, G$. These latent latent variables are then transformed to dummies by:

$$x_{ij} = \begin{cases} 1 & \text{if } z_g \in (q_i, q_{i+1}), \\ 0 & \text{otherwise}, \end{cases}$$

where the q_i are the 0, 1/3, 2/3, and 1th quantiles of the normal distribution.

The second scenario contains many groups of features and is geared towards the group lasso and its extensions. These methods are able to incorporate the grouping of the features, and were originally designed for small interpretable groups, such as dummies of a categorical variable. `gren` on the other hand, relies on large groups of features to accurately estimate group-specific penalty weights. The estimation of many penalty weights in small groups is inefficient and computationally challenging. We therefore expect `gren` to under-perform compared to the other methods.

15.3 Scenario 3

In this scenario we sample the 1000 model parameters in $G = 5$ groups of 200. For each group we generate the parameters in two steps. First we draw 200 parameters from the elastic net distribution, parametrised as in Friedman et al. (2010), where $\alpha = 0.5$ and $\lambda = 100$. The second step consists of setting the (in absolute value) smallest 100 parameters in a group to zero. This results in a total of 500 zero and 500 non-zero parameters, evenly distributed over the five groups. We sample the features from a multivariate normal distribution with all variances 1 and all covariances 0.7.

This third scenario is modelled by considering a situation in which there is no differential signal between the groups, but the correlation within groups is very high. We conjecture that this leads to unstable estimation of the penalty multipliers by `gren` and consequently, performance will suffer.

15.4 Scenario 4

This scenario is similar to Scenario 3, except that we apply differential shrinkage over the groups with penalty multipliers: $\lambda' \in \{0.14, 0.51, 1.95, 7.39\}$. Furthermore, we change the covariance matrix of the predictors to a block diagonal, with block sizes 25×25, such that each group of 200 features consists of 8 blocks of correlated features. The covariance off-diagonals and diagonal in each block are set to $\rho = 0.7$ and $\sigma^2 = 1$, respectively. We expect `gren` to pick up this differential signal and outperform the other methods.

15.5 Scenario 5

In this last scenario, we create $G = 10$ groups of 100 features. Of the 10 groups 8 do not contain any signal. In the two active groups, we set 85 model parameters to zero and 15 to 0.2 and 0.5, respectively, such that the mean signal $\overline{\beta}_j = 21/2000$. The features are simulated from the same distribution as in Scenario 1.

This sparse truth with differential signal between the groups is expected to yield a somewhat better performance by `gren` than the regular elastic net and `GRridge`. The group lasso extensions are expected to give a similar performance.

15.6 Additional results

Results for different values of the tuning parameter α than presented in MD Section 5.1 are given in Figures 20- 24. Overall, these results follow the same pattern as the ones presented in MD Section 5.1.

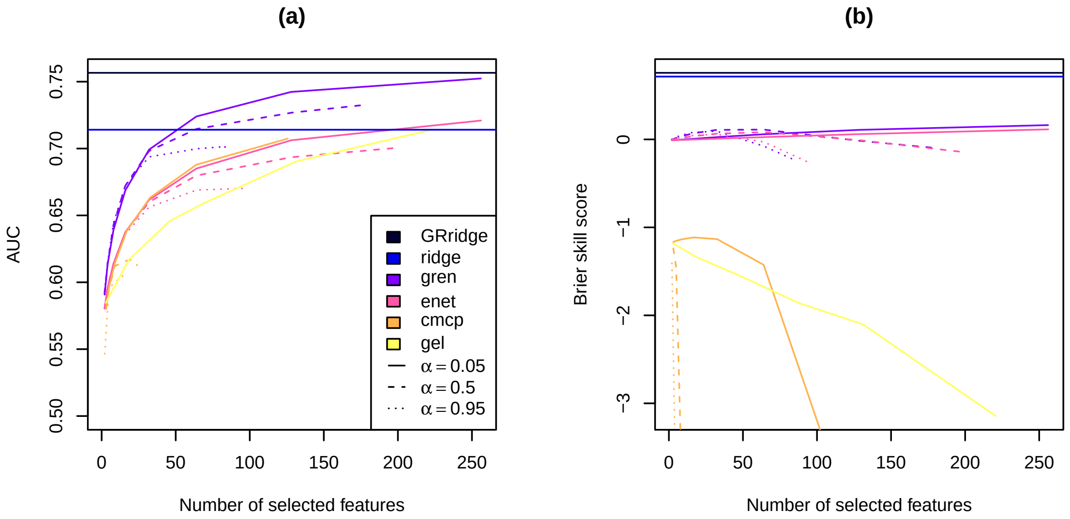

Figure 20: Estimated (a) AUC and (b) Brier skill score in simulation setting 1.

15.7 Model misspecification

To assess the effects of model misspecification we conducted a series of simulations under different modes of misspecification and simulation settings. We investigate model misspecification

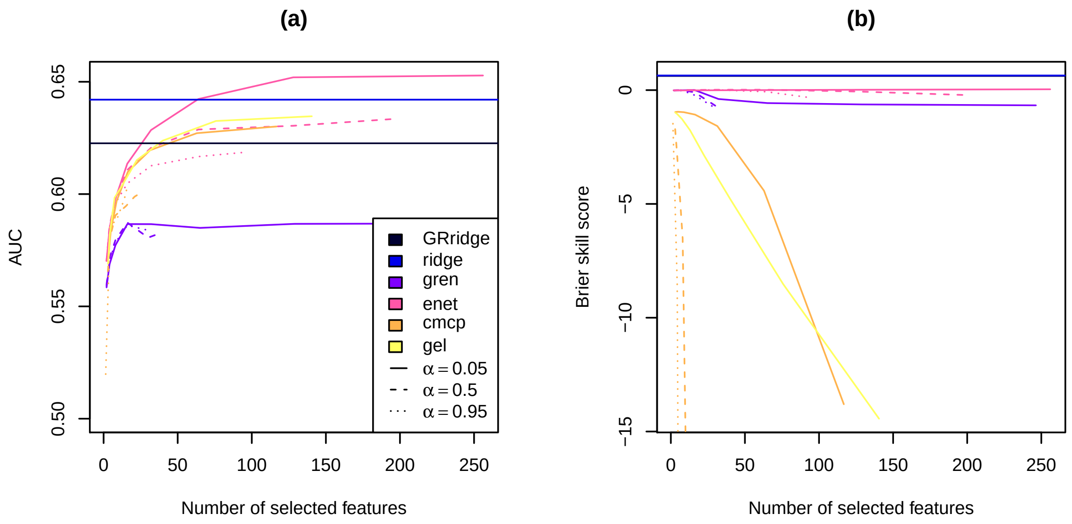

Figure 21: Estimated (a) AUC and (b) Brier skill score in simulation setting 2.

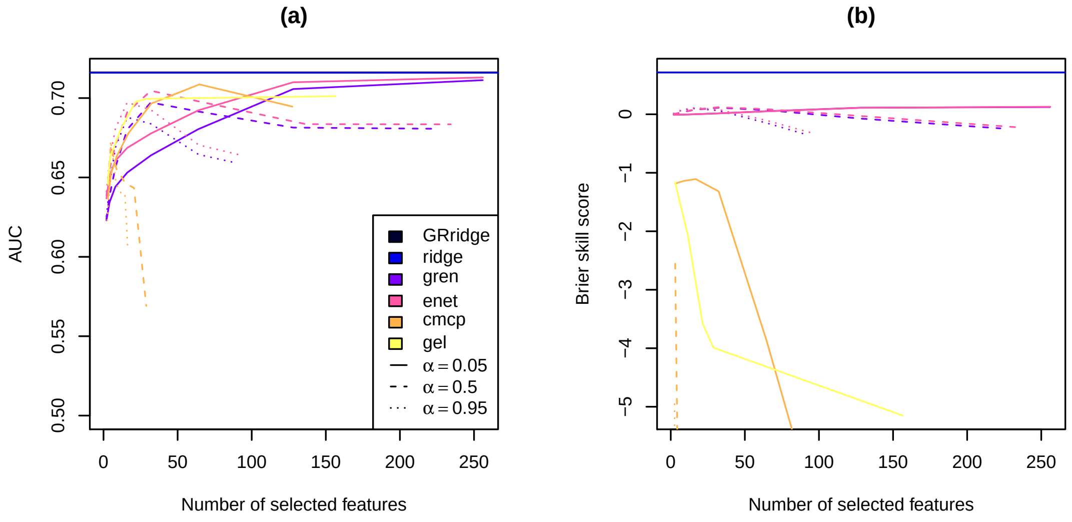

Figure 22: Estimated (a) AUC and (b) Brier skill score in simulation setting 3.

by comparing `gren` to the regular elastic net. To investigate the effect of misspecifying the link function, we simulated according to Scenarios 4 and 5, where we changed the response simulation: $y_i \sim \mathcal{B}(1, [\exp(\mathbf{x}_i^{\mathrm{T}}\boldsymbol{\beta}) + \kappa]/[\exp(\mathbf{x}_i^{\mathrm{T}}\boldsymbol{\beta}) + 1 + \kappa])$ as in Komori et al. (2016), where the link function is unbalanced for $\kappa \neq 0$. We use $\kappa \in \{0.1, 0.5, 1\}$, which leads to 6 different simulations from Scenarios 4 and 5 in total. We present the results in Figures 25- 30. From the Figures we see that `gren` outperforms the regular elastic net even with a misspecified link function in terms of AUC. For the Brier skill score there seems to be some detrimental effect of the misspecification.

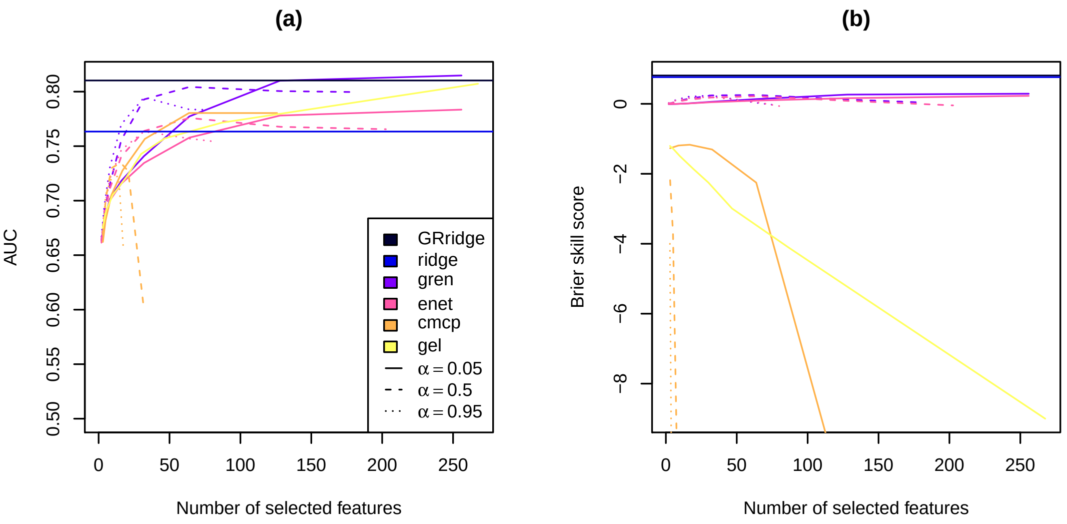

Figure 23: Estimated (a) AUC and (b) Brier skill score in simulation setting 4.

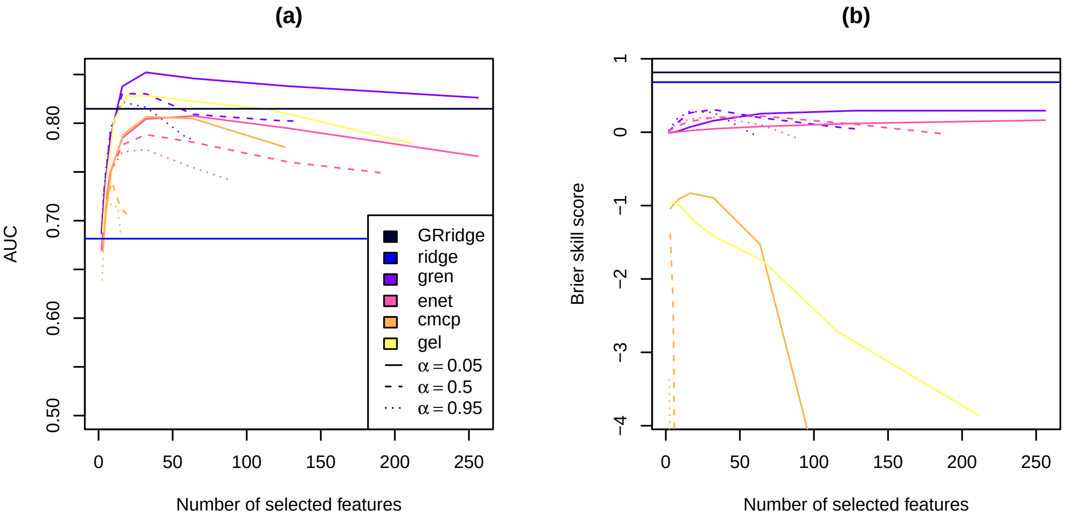

Figure 24: Estimated (a) AUC and (b) Brier skill score in simulation setting 5.

In addition to misspecification of the response relation, model misspecification may also occur in the feature relation. To investigate this we simulated data that includes non-linear relations between linear predictor and features. To do so, we considered the following link function for part of the features: expit$[\max(0, x_{ij})\beta_j]$. We apply this non-linear relation in simulation Scenarios 4 and 5, where we consider three fractions of features for which we apply the non-linear relation: 0.2, 0.4, and 0.6, evenly divided over the groups of features. Meaning that 0.8, 0.6, and 0.4 of the features are linearly related to the link function, respectively. This leads again to 6 different simulation settings: three in Scenario 4 and three in Scenario 5. We present the results in Figures

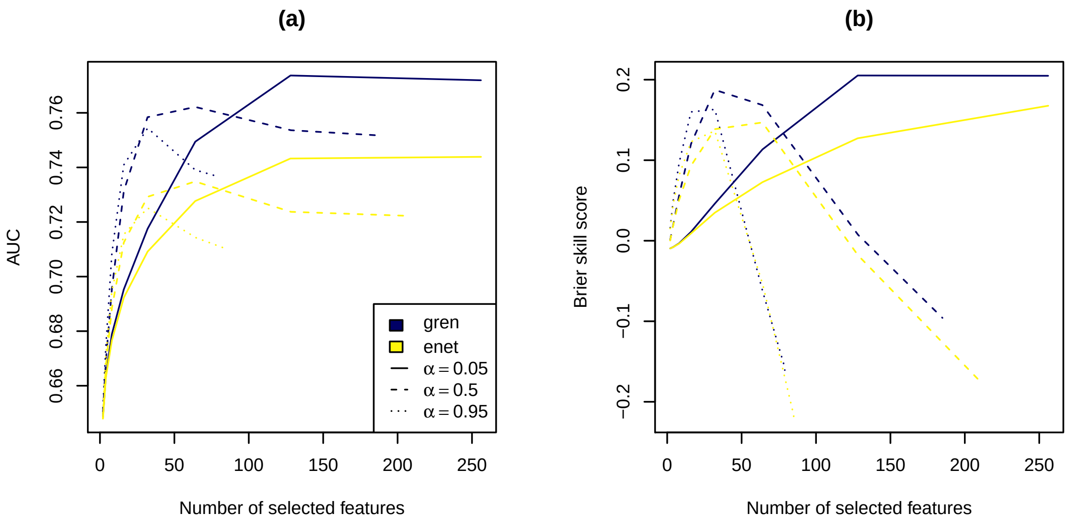

Figure 25: Estimated (a) AUC and (b) Brier skill score in simulation setting 4 for a misspecified link function with $\kappa = 0.1$.

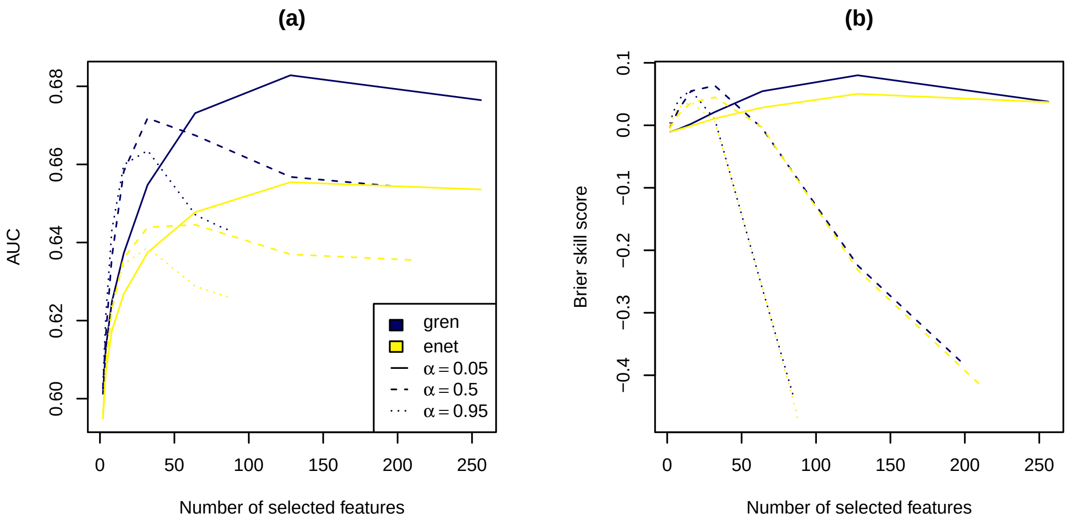

Figure 26: Estimated (a) AUC and (b) Brier skill score in simulation setting 4 for a misspecified link function with $\kappa = 0.5$.

31- 36. From the Figures we see that `gren` still outperforms the regular elastic net, albeit by a slightly smaller margin then with a correctly specified feature relation.

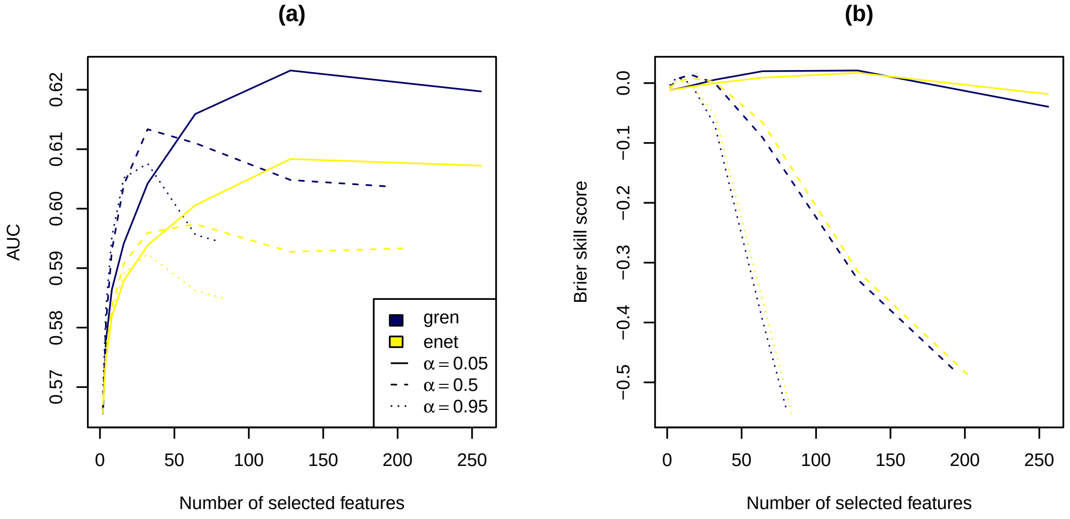

Figure 27: Estimated (a) AUC and (b) Brier skill score in simulation setting 4 for a misspecified link function with $\kappa = 1$.

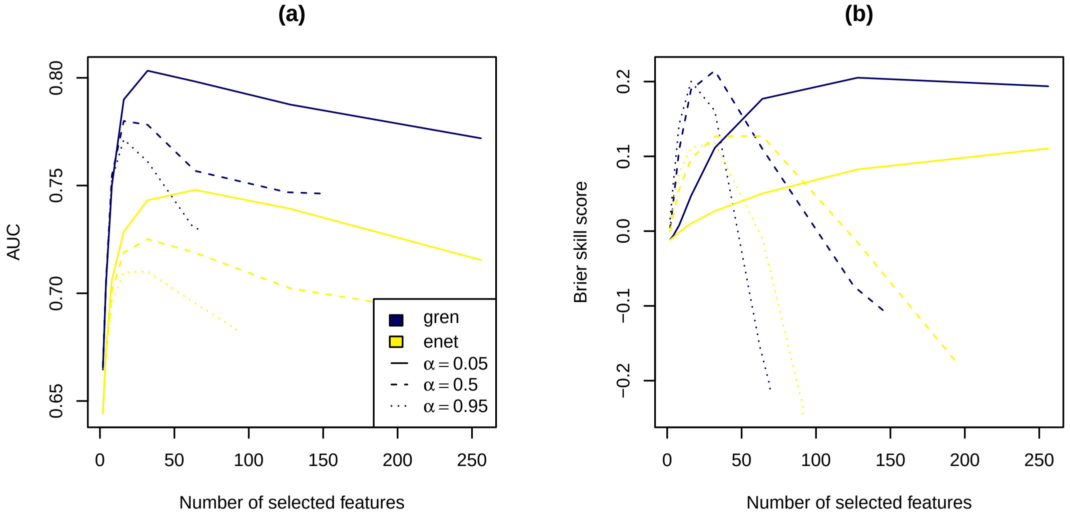

Figure 28: Estimated (a) AUC and (b) Brier skill score in simulation setting 5 for a misspecified link function with $\kappa = 0.1$.

References

Breheny, P. (2015). The group exponential lasso for bi-level variable selection. *Biometrics*, **71**, 731–740.

de Leeuw, F.A. et al. (2017). Blood-based metabolic signatures in Alzheimer's disease. *Alzheimer's*

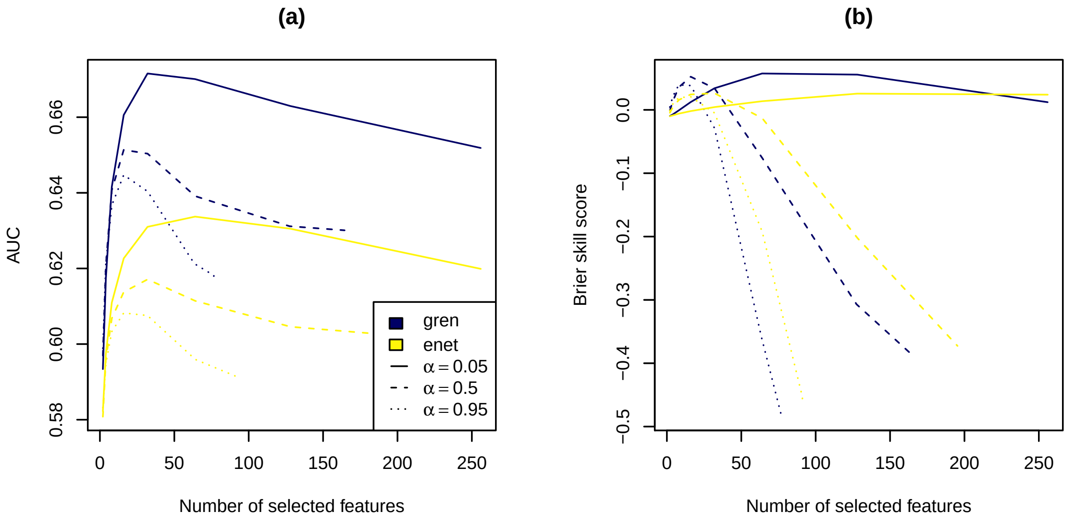

Figure 29: Estimated (a) AUC and (b) Brier skill score in simulation setting 5 for a misspecified link function with $\kappa = 0.5$.

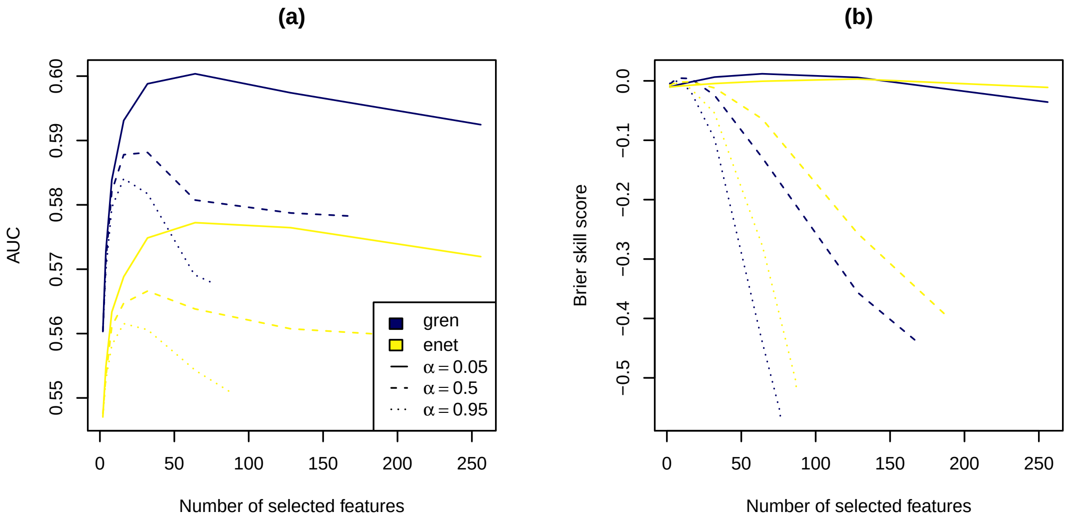

Figure 30: Estimated (a) AUC and (b) Brier skill score in simulation setting 5 for a misspecified link function with $\kappa = 1$.

& Dementia: Diagnosis, Assessment & Disease Monitoring, **8**, 196–207.

Friedman, J. et al. (2010). Regularization paths for generalized linear models via coordinate descent. *Journal of Statistical Software*, **33**, 1–22.

Hastie, T. et al. (2009). *The elements of statistical learning: data mining, inference, and prediction.* Springer series in statistics. Springer, New York, NY, 2nd ed edition.

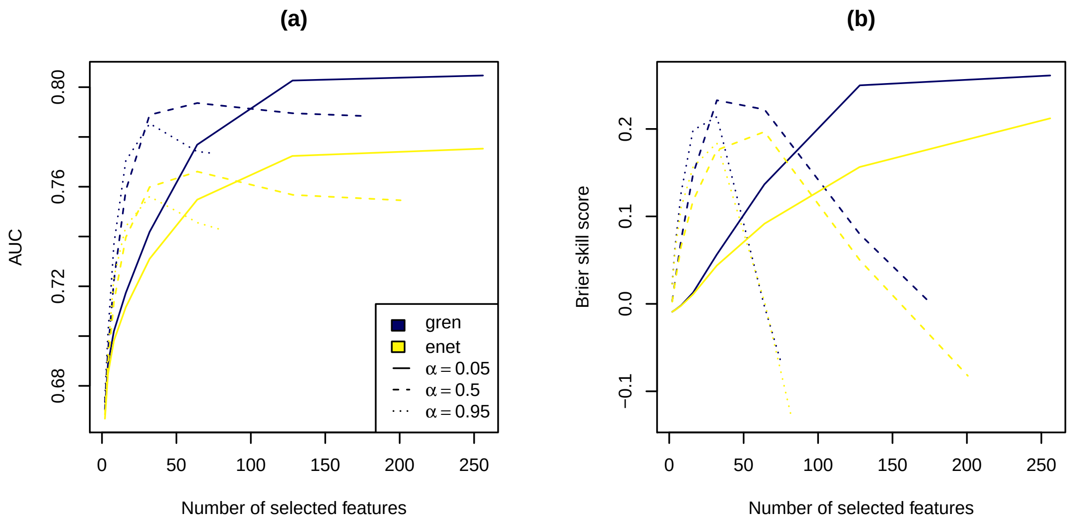

Figure 31: Estimated (a) AUC and (b) Brier skill score in simulation setting 4 for a non-linear feature relation with the fraction of non-linear relations 0.2.

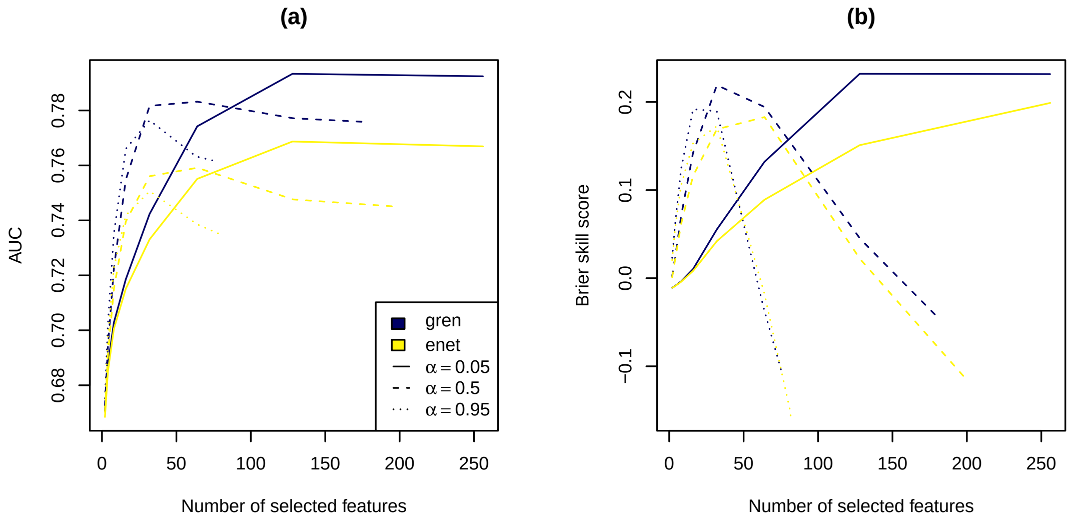

Figure 32: Estimated (a) AUC and (b) Brier skill score in simulation setting 4 for a non-linear feature relation with the fraction of non-linear relations 0.4.

Komori, O. et al. (2016). An asymmetric logistic regression model for ecological data. *Methods in Ecology and Evolution*, **7**, 249–260.

Li, Q. and Lin, N. (2010). The Bayesian elastic net. *Bayesian Analysis*, **5**, 151–170.

Meier, L. et al. (2008). The group lasso for logistic regression: Group Lasso for Logistic Regression. *Journal of the Royal Statistical Society: Series B (Statistical Methodology)*, **70**, 53–71.

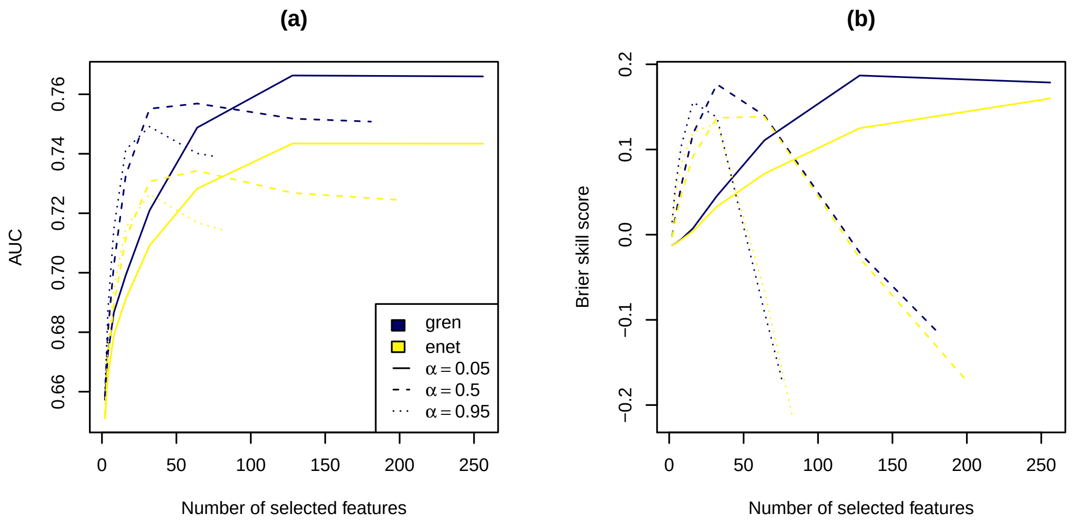

Figure 33: Estimated (a) AUC and (b) Brier skill score in simulation setting 4 for a non-linear feature relation with the fraction of non-linear relations 0.6.

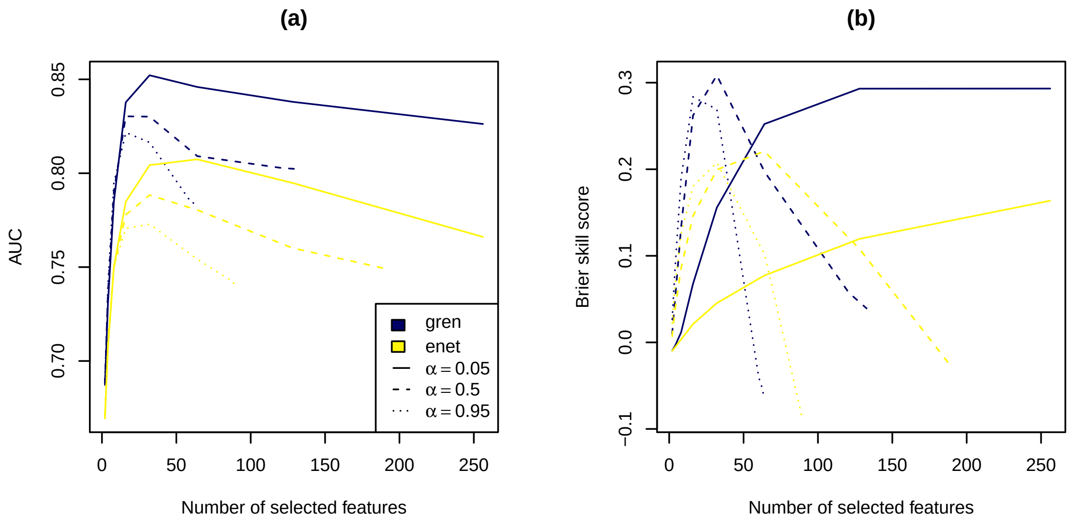

Figure 34: Estimated (a) AUC and (b) Brier skill score in simulation setting 5 for a non-linear feature relation with the fraction of non-linear relations 0.2.

Neerincx, M. et al. (2018). Combination of a six microRNA expression profile with four clini-copathological factors for response prediction of systemic treatment in patients with advanced colorectal cancer. *PLOS ONE*, **13**, e0201809.

Novianti, P.W. et al. (2017). Better diagnostic signatures from RNAseq data through use of auxiliary co-data. *Bioinformatics*, **33**, 1572–1574.

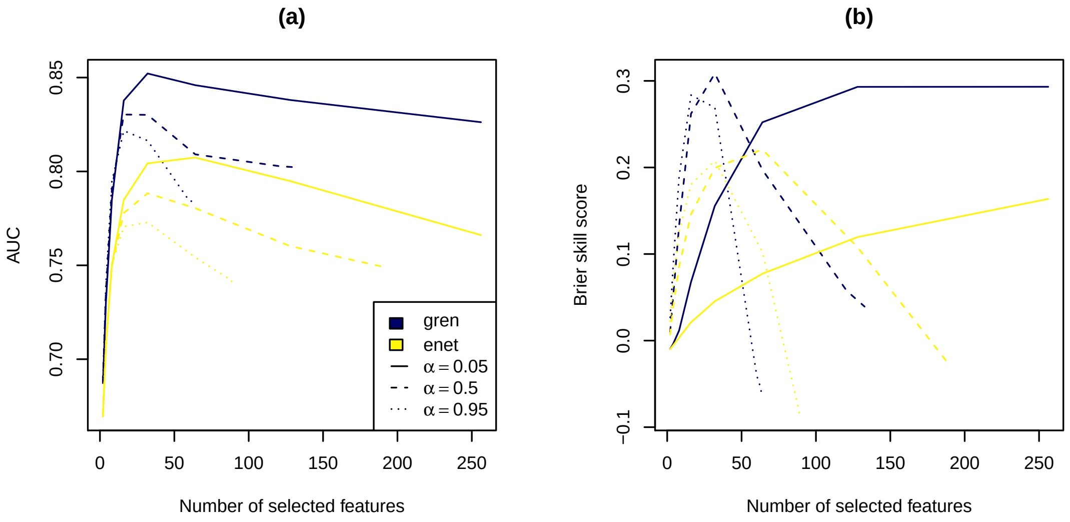

Figure 35: Estimated (a) AUC and (b) Brier skill score in simulation setting 5 for a non-linear feature relation with the fraction of non-linear relations 0.4.

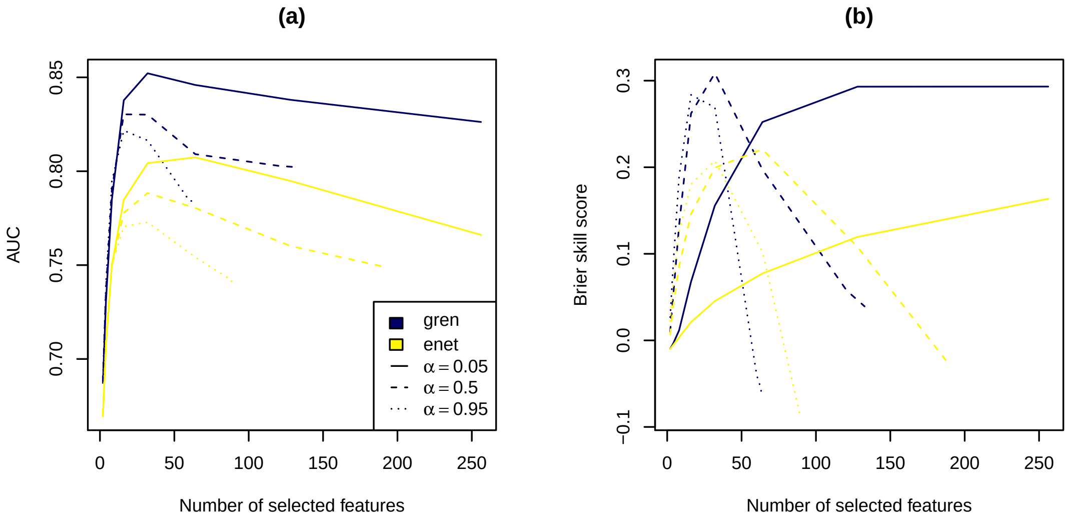

Figure 36: Estimated (a) AUC and (b) Brier skill score in simulation setting 5 for a non-linear feature relation with the fraction of non-linear relations 0.6.

Polson, N.G. et al. (2013). Bayesian Inference for Logistic Models Using Pólya–Gamma Latent Variables. *Journal of the American Statistical Association*, **108**, 1339–1349.

Simon, N. et al. (2013). A Sparse-Group Lasso. *Journal of Computational and Graphical Statistics*, **22**, 231–245.
